# Exercise‐related microRNAs in *Caenorhabditis elegans* regulate calcium homeostasis and mitochondrial dynamics: Conserved pathways, divergent microRNAs

**DOI:** 10.1111/febs.70502

**Published:** 2026-03-30

**Authors:** Qin Xia, Jiwang Tang, José C. Casas‐Martinez, Penglin Li, Maria Borja‐Gonzalez, Leo R Quinlan, Katarzyna Goljanek‐Whysall, Brian McDonagh

**Affiliations:** ^1^ Department of Orthopedics, Tongji Hospital, Tongji Medical College Huazhong University of Science and Technology Wuhan China; ^2^ Discipline of Physiology, School of Pharmacy and Medical Sciences University of Galway Ireland; ^3^ Cellular Physiology Research Laboratory University of Galway Ireland; ^4^ Galway RNA Cluster University of Galway Ireland; ^5^ Institute of Lifecourse and Medical Sciences University of Liverpool UK

**Keywords:** *Caenorhabditis elegans*, DEAD‐box helicase 3 X‐linked (Ddx3x), exercise, inositol polyphosphate‐5‐phosphatase (Inpp5a), Kinase Suppressor of Ras (Ksr1)/*ksr‐2*, microRNA arm switching, peroxiredoxin 2

## Abstract

Exercise can help mitigate age‐related muscle atrophy, promoting mitochondrial function, Ca^2+^ homeostasis and regulating gene expression. MicroRNAs (miRs) are crucial post‐transcriptional regulators of gene expression, fine‐tuning protein levels to maintain cellular homeostasis. In *Caenorhabditis elegans*, a 5‐day swimming regimen increased mitochondrial content, lifespan and fitness. Small RNA sequencing identified exercise specific miRs, including increased levels of cel‐miR‐57‐5p and cel‐miR‐249‐3p, and decreased levels of cel‐miR‐72‐3p and cel‐miR‐77‐5p. *mir‐57* and *mir‐249* mutant strains had enhanced fertility, survival and lifespan, whereas *mir‐72* and *mir‐77* mutant strains had diminished fertility, survival and lifespan. The exercise‐related miRs identified in *C. elegans* did not have conserved mammalian orthologs. Exercise regulated mammalian miRs were identified from the literature including mmu‐miR‐181a‐5p, mmu‐miR‐199a‐5p and mmu‐miR‐378a‐3p. Treatment of murine myoblasts with mmu‐miR‐181a‐5p and mmu‐miR‐378a‐3p enhanced mitochondrial content, autophagy markers and myogenesis, while mmu‐miR‐199a‐5p impaired these processes. Exercise‐related miRs identified in *C. elegans* target genes regulating Ca^2+^ homeostasis such as *ipp‐5* (inositol Polyphosphate‐5‐phosphatase), *sca‐1* (Ca^2+^ transporting ATPase) and *ncx‐2* (mitochondrial Na^+^/Ca^2+^ antiporter). It was also confirmed that mmu‐miR181a‐5p targets Inpp5a and treatment with antogmiR‐181a decreased Ca^2+^ handling in myotubes. Similarly, mmu‐miR‐378a‐3p and cel‐miR‐249‐3p target Kinase Suppressor of Ras (Ksr1)/*ksr‐2*, involved in the MAPK pathway. Despite direct conservation of exercise‐related miRs from nematodes to mammals, there are putative common regulatory pathways contributing to exercise‐induced adaptations.

## Graphical abstract

Exercise‐related microRNAs cel‐miR‐249‐3p and cel‐miR‐77‐5p in *C. elegans* regulate lifespan, fitness, mitochondrial morphology and membrane potential. Although lacking direct mammalian orthologs, H_2_O_2_‐induced microRNAs mmu‐miR‐181a‐5p and mmu‐miR‐378a‐3p regulate myogenesis, autophagy, mitochondrial content and respiration in murine myoblasts. Mechanistically, cel‐miR‐77‐5p and mmu‐miR‐181a‐5p target *ipp‐5*/Inpp5a to coordinate Ca^2+^ homeostasis, while cel‐miR‐249‐3p and mmu‐miR‐378a‐3p target *ksr‐2*/Ksr1 to modulate MAPK signalling. Despite divergent sequences, these microRNAs converge on conserved pathways mediating exercise‐induced muscle adaptation.

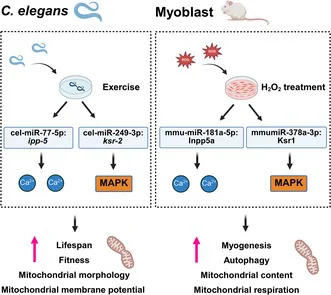

**Abbreviations**
AMPK: AMP‐activated protein kinase
AS: arsenite
ATG5: autophagy related 5
Atp2a1: ATPase sarcoplasmic/endoplasmic reticulum Ca^2+^ transporting 1
CeleST: *C. elegans* swim test
DDX3X: DEAD‐box helicase 3 X‐linked
DMEM: Dulbecco's modified Eagle medium
DTT: Dithiothreitol
FCCP: carbonyl cyanide‐p‐trifluoromethoxyphenylhydrazone
FDR: false discovery rate
FOXO3a: Forkhead box O3a
FWHM: full‐width half‐maximum
HBSS: Hank's Balanced Salt Solution
INPP5A/Inpp5a: inositol polyphosphate‐5‐phosphatase A
IP₃: inositol 1,4,5‐trisphosphate
IPP‐5: inositol polyphosphate‐5‐phosphatase
KIN‐1: cAMP‐dependent protein kinase catalytic subunit
KSR‐1/Ksr1: Kinase Suppressor of Ras 1
KSR‐2: Kinase Suppressor of Ras 2
LAF‐1: Lethal And Fertile 1
LMP‐1: lysosomal membrane protein 1
MAPK/ERK: mitogen‐activated protein kinase/Extracellular signal‐regulated kinase
MAX‐2: serine/threonine‐protein kinase max‐2
MERCS: mitochondrial ER contact sites
MF20: myosin heavy chain antibody
MyoD: myogenic differentiation 1
MyoR: myogenic repressor
NCX‐2: Na/Ca exchanger
NF‐κB: nuclear factor kappa‐light‐chain‐enhancer of activated B cells
NGM: nematode growth medium
NRF2: nuclear factor erythroid 2‐related factor 2
P2Y2R: Purinergic Receptor P2Y2
Pak1: p21‐activated kinase 1
PGC‐1α: peroxisome proliferator‐activated receptor gamma coactivator‐1 alpha
PQ: Paraquat
PRDX‐2: Peroxiredoxin 2
PRKACA: Protein Kinase cAMP‐Activated Catalytic Subunit Alpha
PUF‐9: pumilio homologue 9
SARFIA: semi‐automated region‐based fluorescence intensity analysis
SCA‐1: Sarco‐Endoplasmic Reticulum Calcium ATPase 1
SCPL‐4: Small C‐terminal domain Phosphatase‐Like phosphatase 4
SIRT1: Sirtuin 1
SKN‐1: Skinhead‐1 (NRF2 ortholog)
SLC8A3: solute carrier family 8 member A3
STAT3: signal transducer and activator of transcription 3
TOM20: translocase of outer mitochondrial membrane 20
ULK1: Unc‐51‐like autophagy activating kinase 1
UNC‐54: UNCoordinated 54 (Myosin 4 ortholog)
VDAC1: voltage‐dependent anion channel 1
WLM: worm lysis mix
WNT: Wingless and Int‐1

## Introduction

Skeletal muscle is a dynamic and adaptable tissue that can respond to exercise by promoting muscle hypertrophy and metabolic efficiency [1], whereas disuse, ageing or disease can result in muscle atrophy [2]. Regular exercise can help maintain muscle mass but also exerts multifaceted beneficial effects in age‐related diseases, including coronary atherosclerosis, osteoarthritis and sarcopenia [3, 4, 5]. Age‐related muscle atrophy presents significant public health challenges due to its association with elevated rates of hospitalisation, loss of independence and mortality [6]. Exercise is an effective approach for maintaining muscle mass and function [7]. Nevertheless, the mechanisms underlying the adaptive response to exercise are yet to be fully understood. Mitochondrial dysfunction is a hallmark of ageing, and impaired mitochondrial function has been described in skeletal muscle with age [8, 9]. Exercise has been demonstrated to promote mitochondrial biogenesis and dynamics in skeletal muscle with enhanced metabolic efficiency [10, 11]. During contractile activity, there is endogenous generation of reactive oxygen species (ROS) [12], resulting in an acute stress response and activation of key signalling pathways required for the beneficial adaptive response to exercise [13]. Redox‐sensitive transcription factors such as NRF2, NF‐κB and FOXO are sensitive to an altered intracellular redox environment and are activated following exercise [14, 15], affecting gene transcription and downstream physiological effects. Redox‐dependent signalling and post‐translational modifications can directly affect proteins involved in excitation–contraction coupling and Ca^2+^ handling [16] but also regulate key signalling pathways involved in mitochondrial dynamics, proteostasis and muscle repair [17, 18].

In response to exercise, the acute activation of redox‐sensitive transcription factors can directly affect the transcriptional response. However, the adaptive response to exercise can be fine‐tuned at the post‐transcriptional level by changes in levels of distinct microRNAs (miRs) [19, 20]. miRs are small noncoding RNAs, approximately 19–25 nucleotides in length, with the first 2–8 nucleotides containing the seed sequence that typically binds to the 3′UTR of target genes [21]. miRs play a pivotal role in the modulation of post‐transcriptional gene expression and are reported to regulate 2/3 of the human genome [22]. miRs can inhibit translation of mRNAs and promote mRNA degradation by loading into RNA induced silencing complexes [22, 23]. Furthermore, one miR can potentially target a range of different genes often within the same signalling pathway, and individual genes can be regulated by distinct miRs, further adding complexity to the system. A number of exercise specific miRs have been identified in a range of models from humans to rodents [20]. Many of these miRs contain binding sites for exercise‐related transcription factors such as NRF2, NF‐κB, FOXO etc but also target these redox‐dependent transcription factors providing a negative feedback loop to regulate antioxidant response, mitochondrial dynamics and Ca^2+^ handling. Potentially this provides a mechanism to fine tune the adaptive response to exercise.

*Caenorhabditis elegans* is an established and powerful whole animal model to investigate cellular signalling pathways. The body wall muscle of *C. elegans* closely resembles the sarcomere of striated muscle in vertebrates and is a valuable model for investigation of muscle maintenance and function [24]. Many of the key intracellular signalling pathways are conserved in *C. elegans* and it has been utilised as a model to determine the adaptive response to exercise [25]. It has been demonstrated that an exercise intervention can increase lifespan, help maintain body wall muscle, promote physical activity and delay the onset of neurodegeneration [26, 27]. Following exercise in *C. elegans*, an acute increase in ROS generation has been demonstrated, including activation of SKN‐1 (NRF2 ortholog) and DAF‐16 (FOXO ortholog), resulting in the promotion of mitochondrial turnover and content [28, 29]. Moreover, it was demonstrated that exercise promotes Mitochondrial ER Contact Sites (MERCS) assembly and mitochondrial remodelling [28]. However, the effects of exercise on miR levels in *C. elegans* have not previously been investigated.

In this study, a 5‐day exercise programme that has been demonstrated to increase mitochondrial content and improve healthspan was performed in *C. elegans* [27]. Small RNA sequencing of miRs, revealed changes in specific miRs following exercise. Specifically, there were increased levels of cel‐miR‐57‐5p and cel‐miR‐249‐3p, whereas cel‐miR‐72‐3p and cel‐miR‐77‐5p decreased in response to exercise. Furthermore *mir‐57* and *mir‐249* mutant strains had enhanced fertility, survival and lifespan, whereas *mir‐72* and *mir‐77* mutant strains had diminished fertility, survival and lifespan. Interestingly both *mir‐77* and *mir‐249* mutant strains exhibited impaired mitochondrial morphology and reduced fitness following exercise. cel‐miR‐77‐5p targets a number of genes regulating Ca^2+^ homeostasis such as *ipp‐5* (inositol Polyphosphate‐5‐phosphatase), *sca‐1* (Ca^2+^ transporting ATPase) and *ncx‐2* (mitochondrial Na^+^/Ca^2+^ antiporter). Although the exercise‐related miRs identified in *C. elegans* were not conserved in mammals. Exercise‐related mammalian miRs identified from the literature: mmu‐miR‐181a‐5p, mmu‐miR‐199a‐5p and mmu‐miR‐378a‐3p, shared targets with the exercise‐related miRs identified in *C. elegans*. The levels of mmu‐miR‐181a‐5p, mmu‐miR‐199a‐5p and mmu‐miR‐378a‐3p increased in response to an acute physiological bolus of H_2_O_2_ in an *in vitro* myoblast model. Treatment of myoblasts with synthetic mmu‐miR‐181a‐5p (miR181) and mmu‐miR‐378a‐3p (miR378) led to enhanced mitochondrial content, autophagy markers and improved myogenesis. Among the shared target genes and pathways were the regulation of Ca^2+^ homeostasis and mitochondrial associated genes. Specifically, the results demonstrate that mmu‐miR‐181a‐5p and cel‐miR‐77‐5p commonly target Inositol Polyphosphate‐5‐Phosphatase A (Inpp5a)/*ipp‐5* that reduces inositol 3 phosphate levels (IP_3_), contributing to the regulation of Ca^2+^ metabolism in muscle. Ca^2+^ imaging was performed in myotubes following treatment with miR181 and AntagomiR‐181a (AM181), revealing decreased Ca^2+^ flux with AM181 and elevated Inpp5a. Furthermore, mmu‐miR‐378a‐3p and cel‐miR‐249‐3p share a putative target, Kinase Suppressor of Ras (Ksr1)/*ksr‐2*, involved in activating the Mitogen‐activated protein kinase (MAPK) pathway and mitochondrial biogenesis. The data indicate inter‐species conservation in potential targets of exercise‐related miRs, providing insights into likely conserved regulatory mechanisms in cellular processes related to Ca^2+^ homeostasis and mitochondrial function.

## Results

### Cel‐miR‐57‐5p and cel‐miR‐249‐3p levels increased but cel‐miR‐72‐3p and cel‐miR‐77‐5p levels decreased following chronic swimming exercise

**Fig. 1:**
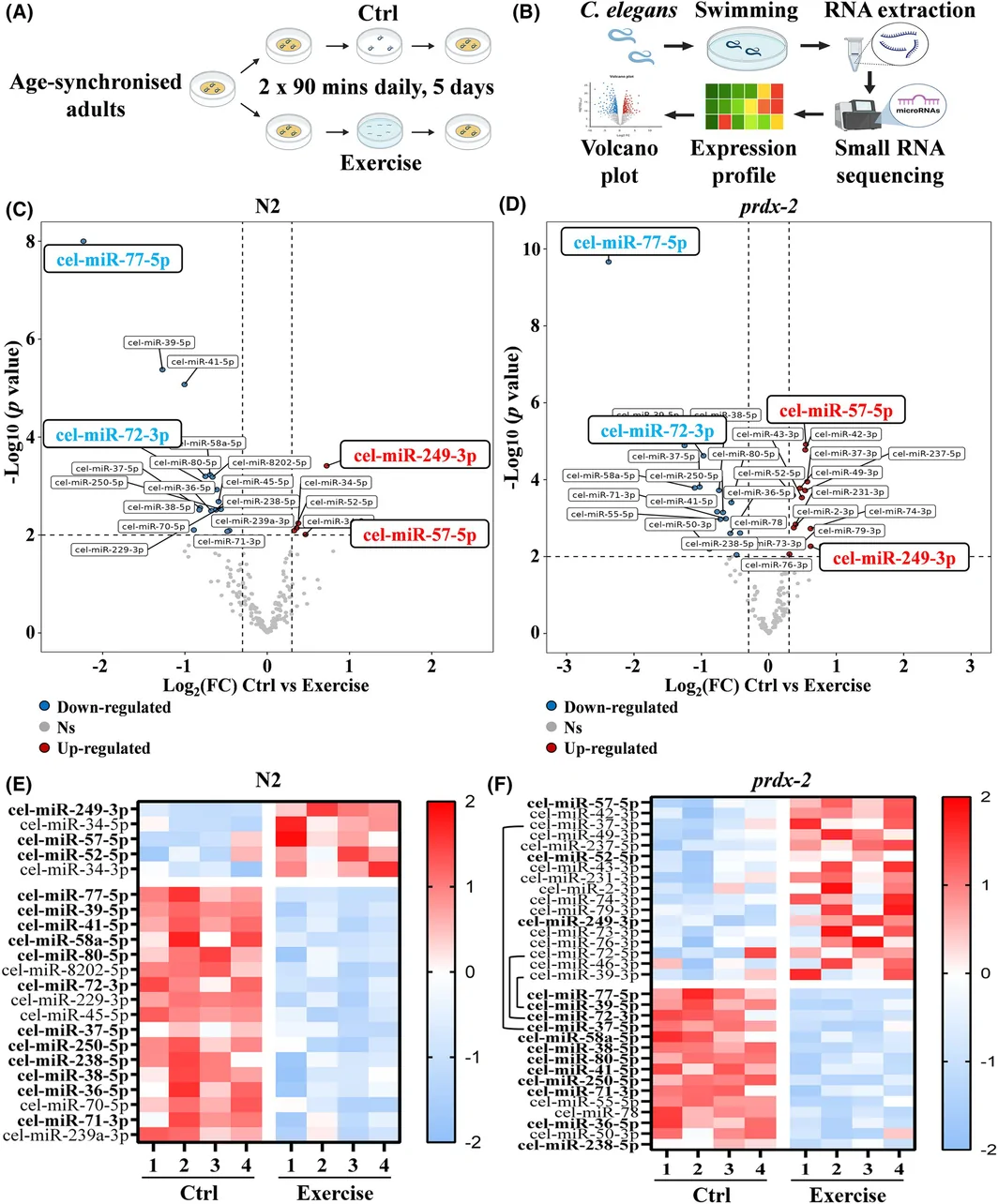
Differential miRs expression in *C. elegans* following a 5‐day swimming protocol. Schematic diagram of the 5‐day swimming protocol in *C. elegans* (A) and processing of samples for small RNA sequencing (B). Differential miRs expression between control and exercised groups in N2 wild‐type strain (FDR 9.0%) (C) and *prdx‐2* mutant strain (FDR 4.5%) (D). Heatmaps of differentially expressed miRs following exercise in N2 (E) and *prdx‐2* mutant strains (F). miRs shown in bold represent those commonly altered in both N2 and *prdx‐2* mutant strains, while those marked with lines indicate arm switching induced by exercise in the *prdx‐2* mutant. Data represent 4 independent replicates per condition. A total of 378 miRs were identified and quantified. miRs with an adjusted *P*‐value <0.01 and a minimum fold change > 1.5 were considered significantly altered by exercise. FDR, false discovery rate.

To identify changes in miRs expression in *C. elegans* in response to exercise, small RNA sequencing was performed following a 5‐day swimming exercise regimen (Fig. 1A,B). The exercise protocol has previously been shown to enhance mitochondrial capacity, improve physical fitness and extend both survival and longevity in wild‐type worms [27]. The exercise regimen resulted in the upregulation of 5 miRs and the downregulation of 17 miRs in the N2 wild‐type strain (Fig. 1C,E). The *prdx‐2* mutant strain has a disrupted response to exercise [27, 28], 17 miRs were upregulated, and 15 miRs were downregulated in this strain (Fig. 1D,F). Interestingly, a previous proteomic analysis in the *prdx‐2* mutant strain identified disrupted microRNA processing following exercise [27], miR‐37, miR‐39, and miR‐72 exhibited opposing changes in the levels of ‐3p and ‐5p mature miRs, suggesting arm switching and miR biogenesis defects (Fig. 1D,F). In the N2 wild‐type and *prdx‐2* mutant strains, cel‐miR‐57‐5p and cel‐miR‐249‐3p were upregulated, whereas cel‐miR‐72‐3p and cel‐miR‐77‐5p were downregulated following exercise suggesting their physiological relevance to exercise in *C. elegans* (Fig. 1C–F).

### Cel‐miR‐57‐5p, cel‐miR‐72‐3p, cel‐miR‐77‐5p and cel‐miR‐249‐3p regulate fertility, survival and lifespan in *C. elegans*

**Fig. 2:**
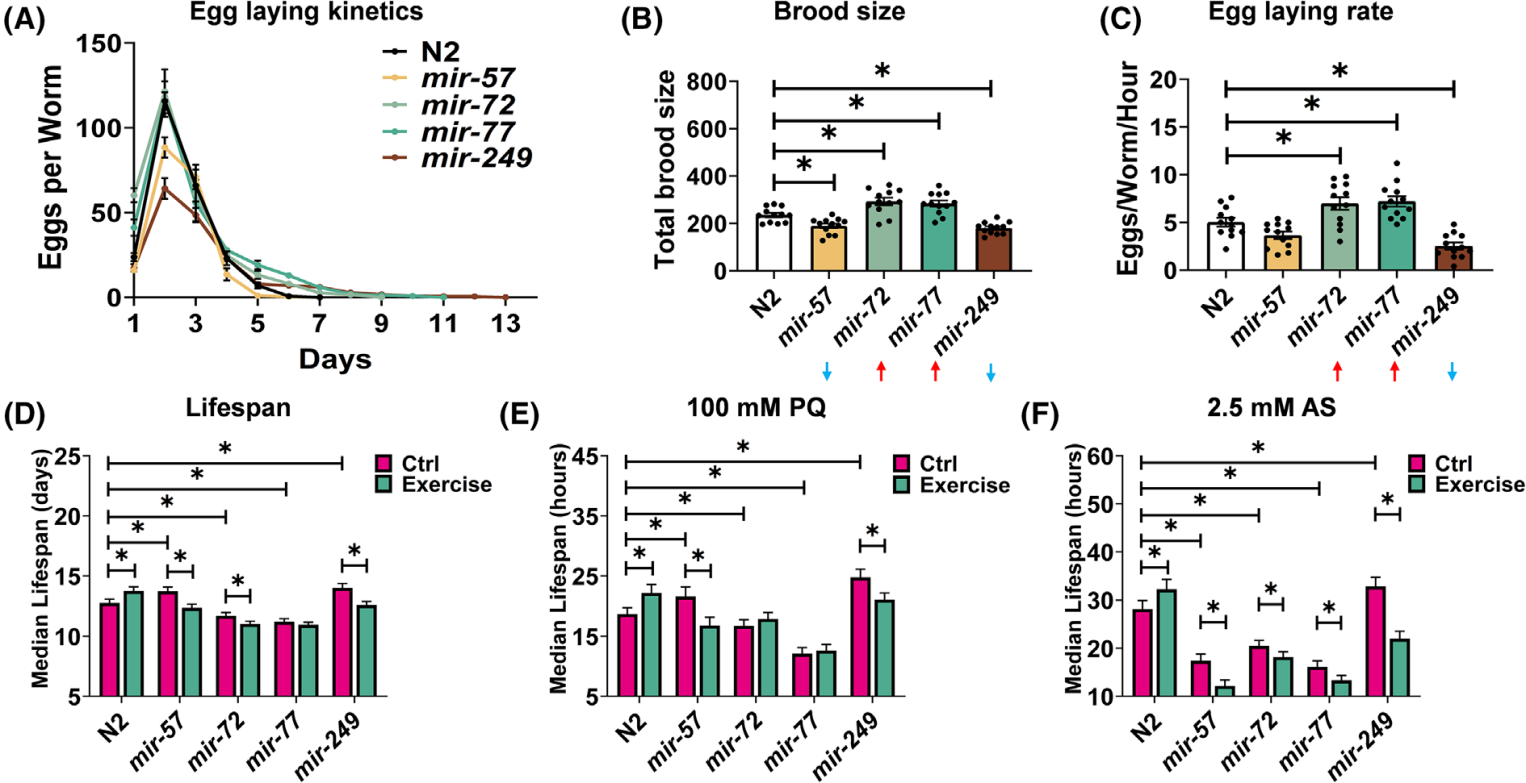
Cel‐miR‐57‐5p, cel‐miR‐72‐3p, cel‐miR‐77‐5p and cel‐miR‐249‐3p regulate fertility, survival and lifespan in *C. elegans*. cel‐miR‐57‐5p, cel‐miR‐72‐3p, cel‐miR‐77‐5p and cel‐miR‐249‐3p regulate fecundity (A–C), lifespan (D) and resistance to oxidative stress following exercise at day 6 (E, F). Graphs are the normalised relative means ± SEM. Experiments were performed with *n* = 12 (egg‐laying assay), *n* = 120 (lifespan assay), *n* = 60 (oxidative stress assay) and *P*‐value of < 0.05 was considered as statistically significant *(*P* < 0.05), one‐way ANOVA (B, C) and log‐rank test (D–F) were used for significance between groups. *P*‐values (B: N2 vs *mir‐57* = 0.0187, N2 vs *mir‐72* = 0.0026, N2 vs *mir‐77* = 0.0133, N2 vs *mir‐249* = 0.0034. C: N2 vs *mir‐72* = 0.0233, N2 vs *mir‐77* = 0.01, N2 vs *mir‐249* = 0.0023. D: N2 vs *mir‐57* = 0.0467, N2 vs *mir‐72* = 0.0103, N2 vs *mir‐77* = 0.0002, N2 vs *mir‐249* = 0.0105; N2: Ctrl vs Exercise = 0.0432; *mir‐57*: Ctrl vs Exercise = 0.0039; *mir‐72*: Ctrl vs Exercise = 0.0364; *mir‐249*: Ctrl vs Exercise = 0.0019. E: N2 vs *mir‐57* = 0.0362, N2 vs *mir‐77* < 0.0001, N2 vs *mir‐249* = 0.0003; N2: Ctrl vs Exercise = 0.0228; *mir‐57*: Ctrl vs Exercise = 0.0144; *mir‐249*: Ctrl vs Exercise = 0.0097. F: N2 vs *mir‐57* < 0.0001, N2 vs *mir‐72* = 0.0002, N2 vs *mir‐77* < 0.0001, N2 vs *mir‐249* = 0.0305; N2: Ctrl vs Exercise = 0.0453; *mir‐57*: Ctrl vs Exercise = 0.0114; *mir‐72*: Ctrl vs Exercise = 0.0402; *mir‐77*: Ctrl vs Exercise = 0.0369; *mir‐249*: Ctrl vs Exercise <0.0001). AS, Arsenite; PQ, paraquat.

To investigate the functional roles of cel‐miR‐57‐5p, cel‐miR‐72‐3p, cel‐miR‐77‐5p and cel‐miR‐249‐3p, we used N2 wild‐type and mutant strains of common miRs upregulated (cel‐miR‐57‐5p and cel‐miR‐249‐3p) and downregulated (cel‐miR‐72‐3p and cel‐miR‐77‐5p) following exercise. Brood size and egg‐laying rates of the strains were assessed. The *mir‐57* and *mir‐249* mutant strains had decreased brood size and egg‐laying rate compared to N2 wild‐type, while the *mir‐72* and *mir‐77* mutant strains had increased brood size (Fig. 2A–C). Subsequently, we performed lifespan and oxidative stress assays including paraquat and arsenite exposure following the swimming exercise. Compared to the N2 nonexercised group, the *mir‐57* and *mir‐249* mutants exhibited an increased lifespan and enhanced resistance to paraquat, while the *mir‐72* and *mir‐77* mutants demonstrated a reduced lifespan and decreased resistance to both paraquat and arsenite (Fig. 2D–F). In the exercised groups, lifespan and survival increased in the N2 wild‐type strain, but decreased in the *mir‐57*, *mir‐72* and *mir‐249* mutants (Fig. 2D,F). These findings underscore the critical roles of cel‐miR‐57‐5p, cel‐miR‐72‐3p, cel‐miR‐77‐5p and cel‐miR‐249‐3p in modulating fertility, survival and lifespan, highlighting their importance in the physiological response to exercise.

### Cel‐miR‐57‐5p, cel‐miR‐72‐3p, cel‐miR‐77‐5p and cel‐miR‐249‐3p influence mitochondrial potential and morphology in *C. elegans*

**Fig. 3:**
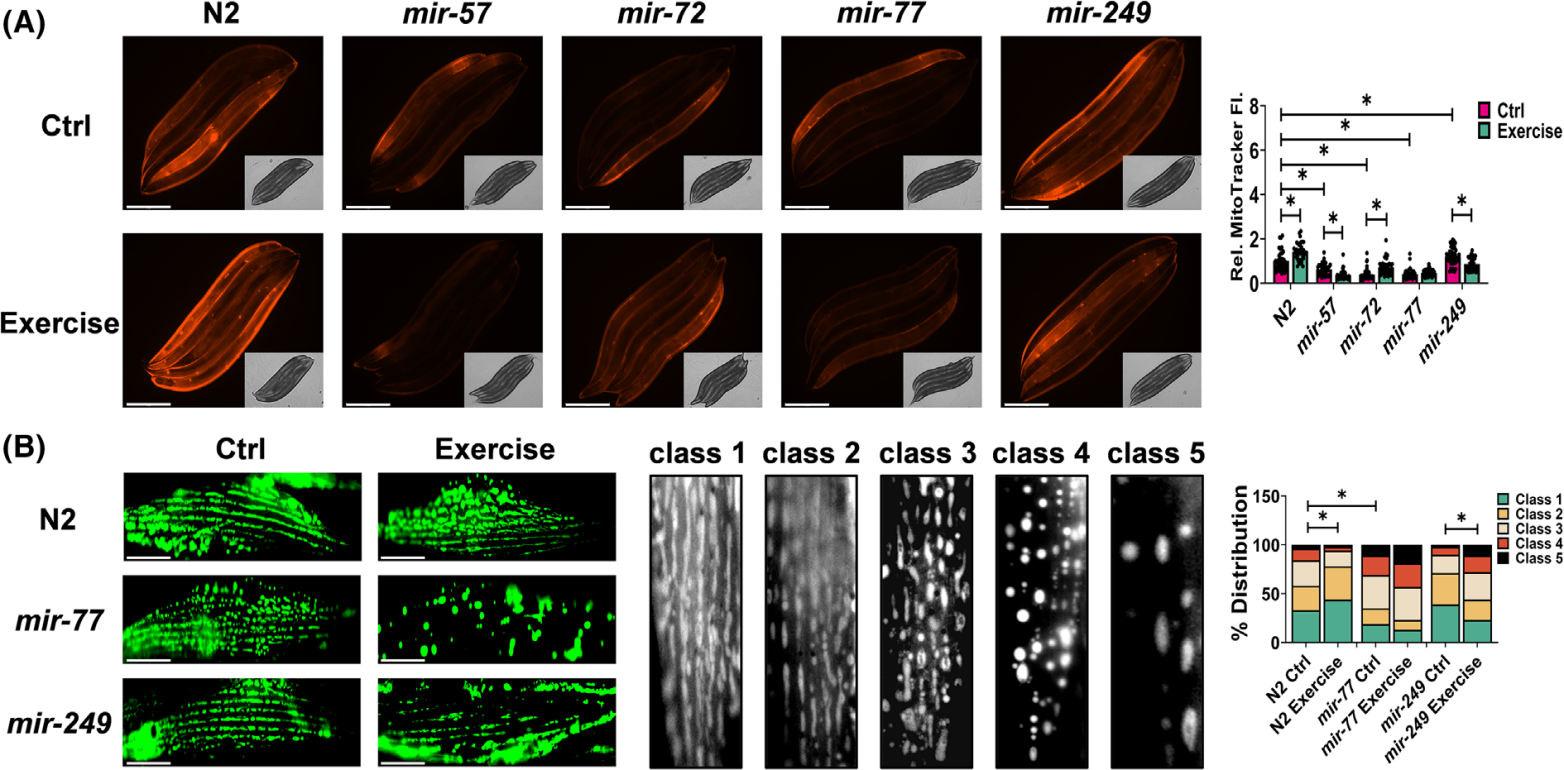
Cel‐miR‐77‐5p and cel‐miR‐249‐3p are required for improved mitochondrial capacity following swimming exercise in *C. elegans*. Representative images of MitoTracker red staining for mitochondrial membrane potential (magenta, scale bar = 275 μm) and *myo‐3::gfp* reporter strain for mitochondrial morphology (green, scale bar = 50 μm) following swimming exercise at day 6 (A, B), mitochondrial morphology were categorised into five distinct classes: class 1, signifying highly abundant and well‐networked mitochondria with minimal or no blebbing; class 2, characterised by highly abundant mitochondria with network gaps and some blebbing; class 3, featuring less abundant mitochondria with network gaps and increased blebbing; class 4, indicating sparse mitochondria with some blebbing; and class 5, denoting very sparse mitochondria. Graphs are the normalised relative means ± SEM. Experiments were performed with *n* = 45 (MitoTracker red staining), *n* = 150 (mitochondria reporter) and *P*‐value of <0.05 was considered as statistically significant *(*P* < 0.05), two‐way ANOVA (A) and chi‐square tests (B) were used for significance between groups. *P*‐values (A: MitoTracker: N2 vs *mir‐57* < 0.0001, N2 vs *mir‐72* < 0.0001, N2 vs *mir‐77* < 0.0001, N2 vs *mir‐249* = 0.0299; N2: Ctrl vs Exercise <0.0001; *mir‐57*: Ctrl vs Exercise = 0.0011; *mir‐72*: Ctrl vs Exercise <0.0001; *mir‐249*: Ctrl vs Exercise <0.0001. B: N2 vs *mir‐77* = 0.0168; N2: Ctrl vs Exercise = 0.0406; *mir‐‐249*: Ctrl vs Exercise = 0.0015).

Given the established link between lifespan, survival ability and mitochondrial function [30], the impact of these miRs on mitochondrial membrane potential and morphology following the swimming exercise was determined. MitoTracker Red staining was used as an indicator of mitochondrial membrane potential. In the nonexercised control groups, the *mir‐249* mutant strain had increased staining intensity compared to N2 wild‐type, while the *mir‐57*, *mir‐72* and *mir‐77* mutants had decreased staining (Fig. 3A). In the exercise group, MitoTracker intensity was enhanced in N2 wild‐type following exercise but decreased in the *mir‐57*, *mir‐72* and *mir‐249* mutant strains. Given the consistent observations in the *mir‐77* and *mir‐249* mutant strains, the mutants were crossed with the muscle mitochondrial reporter strain SJ4103 *zcIs14 [myo‐3p::mitogfp]*. Mitochondrial morphology was quantified based on five distinct categories, each representing a progressive increase in mitochondrial fragmentation and disorganisation [25]. Consistent with the MitoTracker Red staining, the N2 and *mir‐249* mutant strains contained an abundance of interconnected mitochondria in the body wall muscle, whereas the *mir‐77* mutant exhibited increased mitochondrial fragmentation in the body wall muscle (Fig. 3B). Additionally, the swimming exercise increased filamentous mitochondria in the N2 wild‐type strain but resulted in increased mitochondrial fragmentation in both *mir‐77* and *mir‐249* mutants.

### Cel‐miR‐57‐5p, cel‐miR‐72‐3p, cel‐miR‐77‐5p and cel‐miR‐249‐3p influence physical fitness in *C. elegans*

**Fig. 4:**
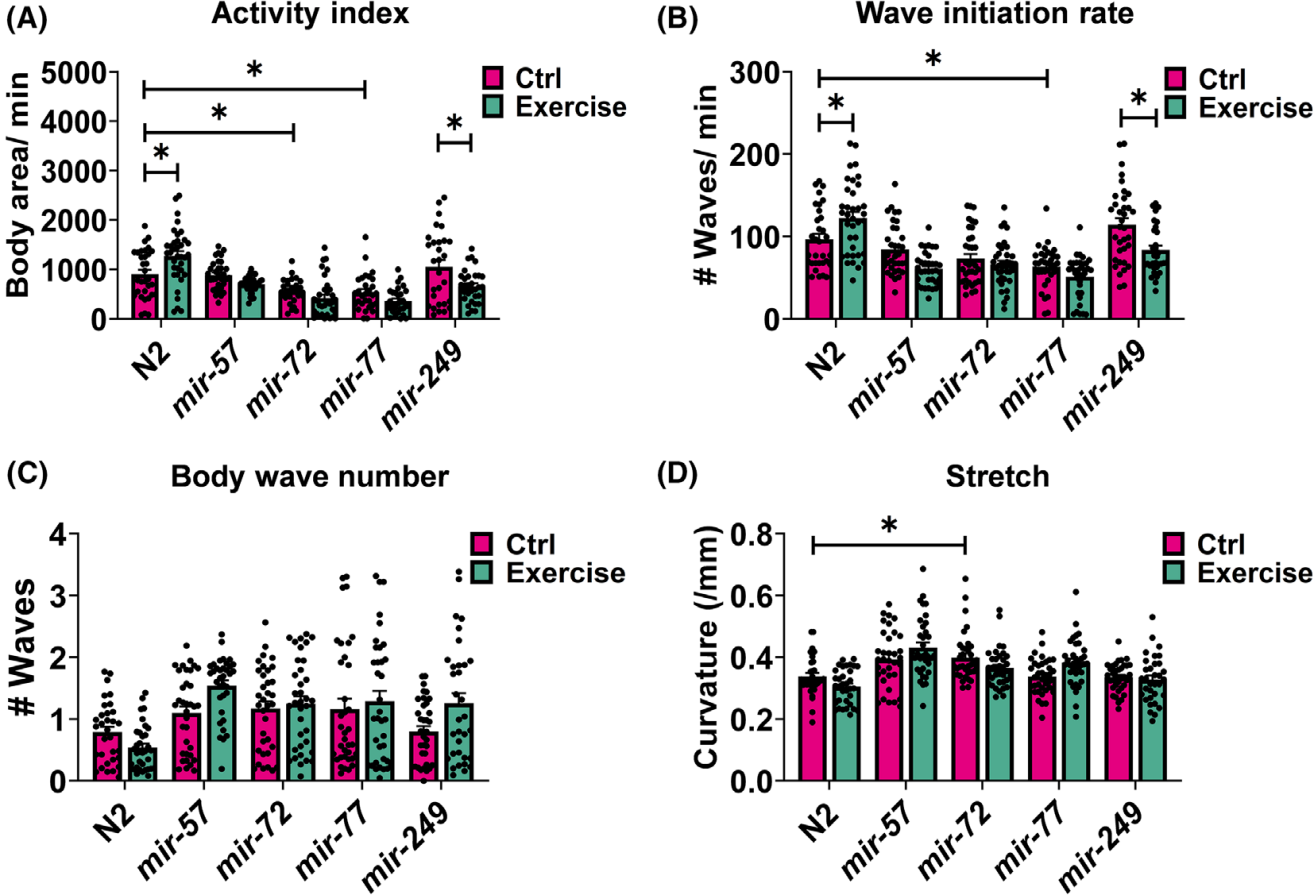
Cel‐miR‐77‐5p and cel‐miR‐249‐3p are required for physical fitness following swimming exercise in *C. elegans*. Physical activity was quantified using CeLeST software for the following parameters: Activity index (A), Wave initiation rate (B), body wave number (C), and stretch (D), following chronic swimming exercise intervention and performed at adult day 6. Graphs are the normalised relative means ± SEM; experiments were performed with *n* = 35 and *P*‐value of <0.05 was considered as statistically significant *(*P* < 0.05), two‐way ANOVA (A–D) was used for significance between groups. *P*‐values (A: N2 vs *mir‐72* = 0.0461, N2 vs *mir‐77* = 0.0179; N2: Ctrl vs Exercise = 0.0112; *mir‐249*: Ctrl vs Exercise = 0.0109. B: N2 vs *mir‐77* = 0.0013; N2: Ctrl vs Exercise = 0.042. D: N2 vs *mir‐72* = 0.0222).

To evaluate the roles of these exercise‐related miRs on physical activity, CeleST was used to track swimming activity parameters [31]. Key metrics such as activity index, wave initiation rate, travel speed and brush stroke were quantified as indicators of enhanced physical fitness, while body wave number, asymmetry, stretch and curling were assessed as markers of frailty [31]. In the nonexercised control groups, the *mir‐77* mutant strain exhibited a decreased activity index and wave initiation rate compared to N2 wild‐type (Fig. 4A,B). Similarly, the *mir‐72* mutant displayed a reduced activity index along with increased stretch (Fig. 4A,D). Following exercise, fitness parameters such as activity index and wave initiation rate increased in the N2 wild‐type (Fig. 4A,B). No changes in body wave number were observed between groups (Fig. 4C). However, the *mir‐249* mutant showed a decreased activity index and wave initiation rate, while no significant changes were observed in the other two mutant strains.

### Mmu‐miR‐181a‐5p, mmu‐miR‐199a‐5p and mmu‐miR‐378a‐3p are upregulated in myoblasts and regulate myogenesis following treatment with physiological levels of H_2_O_2_

A number of exercise‐related miRs have been identified in mammals, regulating a range of physiological functions from mitochondrial turnover to Ca^2+^ homeostasis. When the seed sequences of the miRs identified in *C. elegans* that are responsive to exercise were compared with mammalian miRs, no conserved miRs or seed sequences were identified, highlighting that not all miRs exhibit evolutionary conservation across species [32]. To identify potential conserved signalling pathways, a literature‐based and bioinformatic approach was taken. In mammals it has been previously demonstrated that exercise promotes changes in the intracellular redox environment and activation of redox‐sensitive transcription factors such as NRF2, NF‐κB, FOXO3a and STAT3 [33]. To identify potential miRs regulated by exercise‐related transcription factors, the TransmiR v3.0 database (http://www.cuilab.cn/transmir) was used. A number of miRs were identified that contain binding sites for these redox‐regulated transcription factors including mmu‐miR‐181a‐5p, mmu‐miR‐199a‐5p and mmu‐miR‐378a‐3p (Table 1). NRF2 and STAT3 jointly regulate miR‐181 [34, 35], NF‐κB regulates miR‐199 and miR‐378 [36, 37, 38]. Moreover, these miRs have been previously shown to play crucial roles in the regulatory processes of skeletal muscle myogenesis [39] and in response to exercise (Table 1). miR‐181a‐5p levels increase with exercise and decrease with age [40, 41], it has been reported to regulate key components of mitochondrial turnover and enhances myogenesis through the targeting of Hox‐A11 [42]. miR‐199 inhibits myogenesis by targeting the WNT signalling pathway [43]. miR‐378 regulates myogenesis by targeting MyoR and enhancing MyoD expression [44].

**Table 1:** Selected miRs identified as having a role in exercise

miRs	Function in myogenesis	Expression subject to exercise	Expression regulated by TFs (NRF2, NF‐κB, FOXO3a, STAT3)
miR‐181‐5p	Promotes myogenesis by targeting Hox‐A11 [42]	Exercise enhances the expression of miR‐181 and mitochondrial biogenesis [40]	NRF2 and STAT3 jointly regulate miR‐181 [34, 35]
miR‐199‐5p	Inhibits myogenesis by targeting Fzd4, Jag1, Wnt2 [43]	miR‐199 indicates poor aerobic exercise capacity in response to high altitude [57]	NF‐κB regulates miR‐199 [36, 37]
miR‐378‐3p	Promotes myogenesis by targeting MyoR [44]	Physical exercise increased the expression of miR‐378 [115]	NF‐κB regulates miR‐378 [38]

**Fig. 5:**
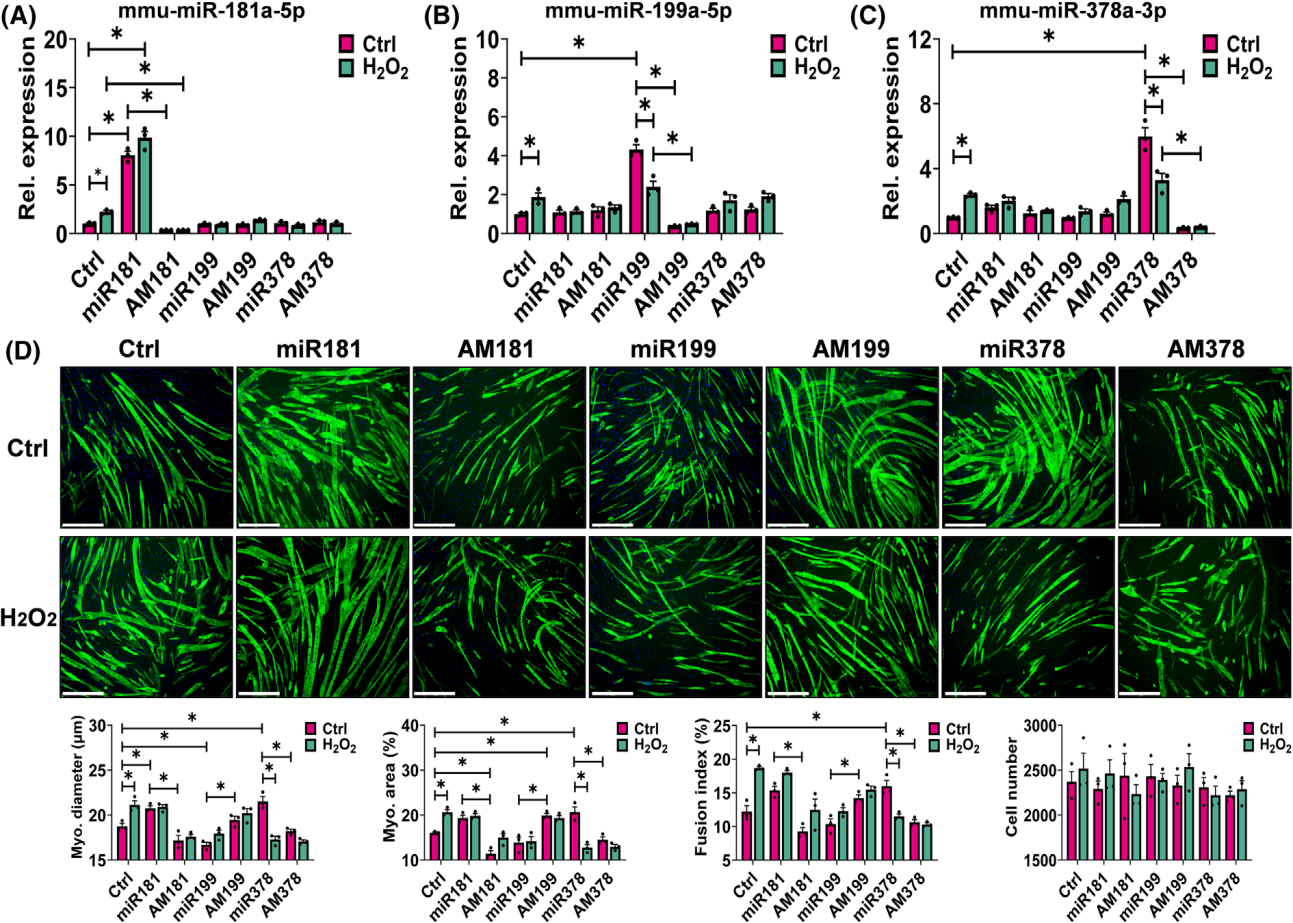
Mmu‐miR‐181a‐5p, mmu‐miR‐199a‐5p and mmu‐miR‐378a‐3p are upregulated following an acute physiological concentration of H_2_O_2_ and influence myogenesis in C2C12 cells. C2C12 cells were transfected with mmu‐miR‐181a‐5p, mmu‐miR‐199a‐5p, mmu‐miR‐378a‐3p mimics and antagomirs following physiological levels of H_2_O_2_ treatment (A–C). MF20 immunostaining of myotubes (green, scale bar = 75 μm) following differentiation of myoblasts treated with selected miRs and their antagomiRs was performed to determine myotube diameter, myotube area, fusion index and cell number (D). Graphs are the normalised relative means ± SEM, experiments were performed with *n* = 3 and *P*‐value of <0.05 was considered as statistically significant *(*P* < 0.05), two‐way ANOVA (A‐D) was used for significance between groups. *P*‐values (A: in control group: ctrl vs miR181 < 0.0001, miR181 vs AM181 < 0.0001; in H_2_O_2_ group: ctrl vs AM181 = 0.0001; Ctrl: ctrl vs H_2_O_2_: ctrl = 0.0264, Ctrl: ctrl vs H_2_O_2_: miR181 < 0.0001. B: in control group: ctrl vs miR199 < 0.0001, miR199 vs AM199 < 0.0001; in H_2_O_2_ group: miR199 vs AM199 < 0.0001; Ctrl: ctrl vs H_2_O_2_: ctrl = 0.0423, Ctrl: ctrl vs H_2_O_2_: miR199 = 0.0001, Ctrl: miR199 vs H_2_O_2_: miR199 < 0.0001. C: in control group: ctrl vs miR378 < 0.0001, miR378 vs AM378 < 0.0001; in H_2_O_2_ group: miR378 vs AM378 < 0.0001; Ctrl: ctrl vs H_2_O_2_: ctrl = 0.0047, Ctrl: miR378 vs H_2_O_2_: miR378 < 0.0001. (D) Myotube diameter: in control group: ctrl vs miR181 = 0.0401, ctrl vs miR199 = 0.0329, ctrl vs miR378 = 0.0011, miR181 vs AM181 < 0.0001, miR199 vs AM199 = 0.0011, miR378 vs AM378 < 0.0001; Ctrl: ctrl vs H_2_O_2_: ctrl = 0.0077, Ctrl: ctrl vs H_2_O_2_: miR378 < 0.0001, Ctrl: miR378 vs H_2_O_2_: miR378 < 0.0001. Myotube area: in control group: ctrl vs AM181 = 0.0073, ctrl vs AM199 = 0.0462, ctrl vs miR378 = 0.0072; miR181 vs AM181 < 0.0001, miR199 vs AM199 = 0.0003, miR378 vs AM378 = 0.0002; Ctrl: ctrl vs H_2_O_2_: ctrl = 0.0076; H_2_O_2_: ctrl vs H_2_O_2_: miR378 < 0.0001; Ctrl: miR378 vs H_2_O_2_: miR378 < 0.0001. Fusion index: in control group: ctrl vs miR378 = 0.0302, miR181 vs AM181 < 0.0001, miR199 vs AM199 = 0.0249, miR378 vs AM378 = 0.0005; Ctrl: ctrl vs H_2_O_2_: ctrl <0.0001, H_2_O_2_: ctrl vs H_2_O_2_: miR378 < 0.0001, Ctrl: miR378 vs H_2_O_2_: miR378 = 0.0049).

To evaluate the redox sensitivity of specific miRs in skeletal muscle, myoblasts were exposed to 25 μm H_2_O_2_ for 10 min and allowed to recover in fresh media for 24 h. This concentration and duration of H_2_O_2_ has previously been shown to increase mitochondrial content and promote myogenesis [27]. qPCR analysis revealed a significant increase in the levels of mmu‐miR‐181a‐5p, mmu‐miR‐199a‐5p and mmu‐miR‐378a‐3p 24 h postexposure to acute H_2_O_2_ (Fig. 5A–C). Given the limited understanding of the roles of mmu‐miR‐181a‐5p, mmu‐miR‐199a‐5p and mmu‐miR‐378a‐3p in regulating mitochondrial capacity and myogenesis in skeletal muscle, this study further explored their function by treating myoblasts with synthetic miR mimics (miR181, miR199, miR378) and antagomirs (AM181, AM199, AM378) prior to H_2_O_2_ exposure. Specifically, the miR181, miR199 and miR378 groups significantly increased, while AM181, AM199 and AM378 groups significantly decreased the levels of the respective miRs (Fig. 5A–C). The qPCR results consistently demonstrated an increase in the expression of these miRs following H_2_O_2_ treatment in the control group (Fig. 5A–C). Additionally, following H_2_O_2_ treatment, mmu‐miR‐199a‐5p and mmu‐miR‐378a‐3p levels decreased in the miR199 and miR378 treated groups (Fig. 5B,C).

To determine the effects of altered miR expression on myogenesis, we performed MF20 staining following differentiation of myoblasts into myotubes. In the control groups, the miR181, AM199 and miR378 treatments resulted in enhanced myotube diameter, myotube area and fusion index (Fig. 5D). However, the groups treated with AM181 and miR199 demonstrated decreased myotube diameter and myotube area. In the H_2_O_2_‐treated control group, there was improved myogenesis; however, in the miR378 group treated with H_2_O_2_, impaired myogenesis was observed. Collectively, these findings suggest that mmu‐miR‐181a‐5p and mmu‐miR‐378a‐3p play crucial roles in promoting myogenesis.

### miR181, AM199 and miR378 increased mitochondrial content and autophagy markers

**Fig. 6:**
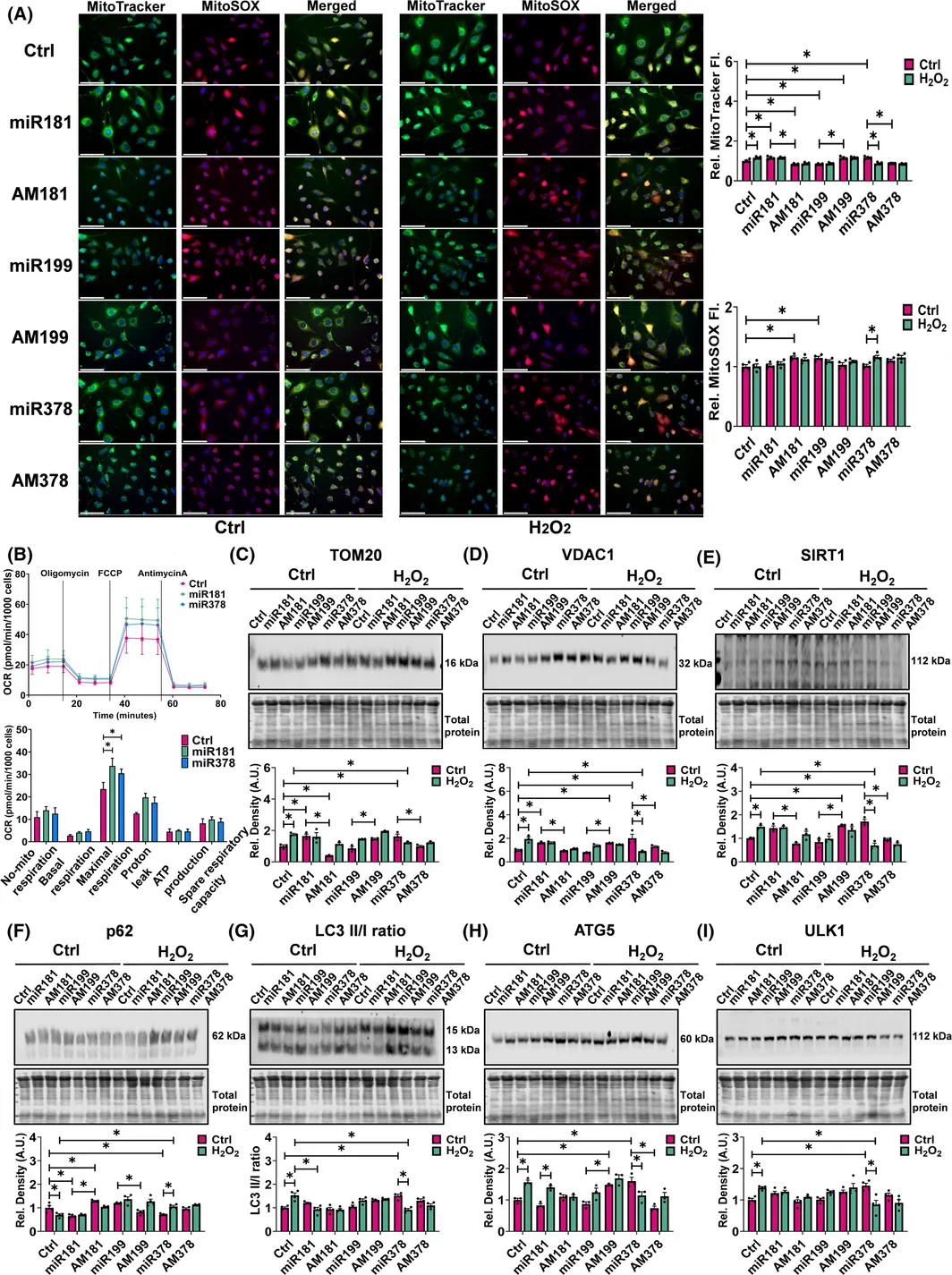
Mmu‐miR‐181a‐5p, mmu‐miR‐199a‐5p and mmu‐miR‐378a‐3p regulate mitochondrial content and autophagy markers in myoblasts. MitoTracker and MitoSOX immunostaining (scale bar = 75 μm) demonstrating changes in mitochondrial content (green) and ROS levels (magenta) (A); Seahorse analysis of oxygen consumption demonstrating mmu‐miR‐181a‐5p and mmu‐miR‐378a‐3p increase maximal respiration (B); Western blots indicating changes in the protein levels of TOM20, VDAC and SIRT1 (C–E), autophagy‐associated proteins p62, LC3 II/I, ATG5 and ULK1 (F–I), lower panel shows Ponceau S stain of total protein on membranes that was used for normalisation. Graphs are the normalised relative means ± SEM, experiments were performed with *n* = 3 and *P*‐value of <0.05 was considered as statistically significant *(*P* < 0.05), two‐way ANOVA (A, C–I) and one‐way ANOVA (B) were used for significance between groups. Western blots for TOM20, VDAC1, SIRT1 and ATG5 were performed using the same nitrocellulose membranes and normalised using the same Ponceau S stains. Similarly, LC3 II/I and p62 Western blots were performed using the same nitrocellulose membranes and normalised to total protein from the same Ponceau S stains. Replicates of original blots and Ponceau S stains can be found in the source data file from Mendeley Data DOI: 10.17632/sj5bstcj4m.1. *P*‐values (A: MitoTracker: in control group: ctrl vs miR181 = 0.0346, ctrl vs AM181 = 0.0196, ctrl vs miR199 = 0.0269, ctrl vs AM199 = 0.0462, ctrl vs miR378 = 0.0229, miR181 vs AM181 < 0.0001, miR199 vs AM199 < 0.0001, miR378 vs AM378 < 0.0001; Ctrl: ctrl vs H_2_O_2_: ctrl = 0.0248, Ctrl: miR378 vs H_2_O_2_: miR378 < 0.0001. MitoSOX: in control group: ctrl vs AM181 = 0.024, ctrl vs miR199 = 0.0418, ctrl vs miR199 = 0.0418; Ctrl: ctrl vs H_2_O_2_: miR378 = 0.0176; Ctrl: miR378 vs H_2_O_2_: miR378 = 0.05). B: in maximal respiration: Ctrl vs miR181 = 0.0022, Ctrl vs miR378 = 0.0398). C: in control group: ctrl vs miR181 = 0.0063, ctrl vs AM181 = 0.0122, ctrl vs miR378 = 0.0103, miR181 vs AM181 < 0.0001, miR199 vs AM199 = 0.0121, miR378 vs AM378 = 0.0088; Ctrl: ctrl vs H_2_O_2_: ctrl = 0.0009; in H_2_O_2_ group: ctrl vs miR378 = 0.0442. D: in control group: ctrl vs miR181 = 0.025, ctrl vs AM199 = 0.0335, ctrl vs miR378 < 0.0001, miR181 vs AM181 = 0.0094, miR199 vs AM199 = 0.0013, miR378 vs AM378 = 0.0066; in H_2_O_2_ group: ctrl vs miR378 < 0.0001; Ctrl: ctrl vs H_2_O_2_: ctrl = 0.0001; Ctrl: miR378 vs H_2_O_2_: miR378 < 0.0001. E: in control group: ctrl vs AM199 = 0.0082, ctrl vs miR378 = 0.0003, miR181 vs AM181 = 0.0008, miR199 vs AM199 = 0.0003, miR378 vs AM378 = 0.0001; Ctrl: ctrl vs H_2_O_2_: ctrl = 0.0282, Ctrl: miR378 vs H_2_O_2_: miR378 < 0.0001. F: in control group: ctrl vs miR181 = 0.0033, ctrl vs AM181 = 0.027, ctrl vs miR378 = 0.0317, miR181 vs AM181 < 0.0001, miR199 vs AM199 = 0.0011; in H_2_O_2_ group: ctrl vs miR378 = 0.0018; Ctrl: ctrl vs H_2_O_2_: ctrl = 0.0163, Ctrl: miR378 vs H_2_O_2_: miR378 = 0.0039. G: in control group: ctrl vs miR378 = 0.0001; Ctrl: ctrl vs H_2_O_2_: ctrl <0.0001; H_2_O_2_: ctrl vs H_2_O_2_: miR181 < 0.0001, H_2_O_2_: ctrl vs H_2_O_2_: miR378 < 0.0001, Ctrl: miR378 vs H_2_O_2_:miR378 < 0.0001. H: in control group: ctrl vs AM199 = 0.0287, ctrl vs miR378 = 0.0025, miR199 vs AM199 = 0.0021, miR378 vs AM378 < 0.0001; Ctrl: ctrl vs H_2_O_2_: ctrl = 0.0053; Ctrl: miR378 vs H_2_O_2_: miR378 = 0.0445. I: in control group: ctrl vs miR378 = 0.0062; Ctrl: ctrl vs H_2_O_2_: ctrl = 0.034; H_2_O_2_: ctrl vs H_2_O_2_: miR378 = 0.0005, Ctrl: miR378 vs H_2_O_2_: miR378 < 0.0001).

The potential regulatory roles of mmu‐miR‐181a‐5p, mmu‐miR‐199a‐5p and mmu‐miR‐378a‐3p on mitochondrial content, ROS and autophagy markers were explored. In order to determine the effects of mmu‐miR‐181a‐5p, mmu‐miR‐199a‐5p and mmu‐miR‐378a‐3p on mitochondria, we performed MitoTracker green staining for mitochondrial content and MitoSOX red staining for mitochondrial ROS, following the acute H_2_O_2_ treatment. In the control groups, the miR181, AM199 and miR378 groups exhibited increased MitoTracker green staining (Fig. 6A) supporting the enhanced myogenesis observed (Fig. 5D). However, the AM181 and miR199 groups demonstrated decreased MitoTracker green staining and increased MitoSOX fluorescence intensity (Fig. 6A), along with decreased myotube diameter and area (Fig. 5D). In the H_2_O_2_‐treated group, myoblasts exhibited heightened mitochondrial content; however, the miR378 group treated with H_2_O_2_ had decreased MitoTracker green intensity and increased MitoSOX intensity (Fig. 6A) and impaired myogenesis (Fig. 5D). The effects of mmu‐miR‐181a‐5p and mmu‐miR‐378a‐3p on mitochondrial respiration in myoblasts were also determined using the Seahorse Bioanalyzer. The miR181 and miR378 groups had increased maximal respiration, although no significant change in basal respiration, suggesting increased respiratory capacity (Fig. 6B).

To support the changes in mitochondria observed by immunostaining, Western blotting was performed for markers of mitochondrial content and autophagy. In the control group, Western blots revealed that the miR181, miR378 and AM199 treatment led to increased levels of mitochondrial associated proteins, including TOM20, VDAC and SIRT1 (Fig. 6C–E). In the H_2_O_2_ treated group, elevated levels of TOM20, VDAC and SIRT1 were also observed. However, a notable reduction in these proteins was observed in the miR378 group following H_2_O_2_ treatment. Previously, it has been reported that mmu‐miR‐181a‐5p and mmu‐miR‐378a‐3p play a role in autophagy regulation in mammalian cells [41, 45]. In response to the acute H_2_O_2_ treatment, the control group had decreased p62 levels, an increased LC3 II/I ratio, and increased ATG5 and ULK1 levels suggesting a promotion of autophagy (Fig. 6F–I). The miR181 group had decreased p62 and increased ATG5 and Ulk1 in response to H_2_O_2_, whereas the AM181 group exhibited increased p62 levels, suggested stalled autophagy (Fig. 6F). Moreover, the miR378 group exhibited an elevated LC3 II/I ratio and increased ATG5 and ULK1, along with a decrease in p62 levels (Fig. 6F–I), Collectively, these findings suggest that mmu‐miR‐181a‐5p, mmu‐miR‐199a‐5p and mmu‐miR‐378a‐3p play crucial roles in regulating mitochondrial content, autophagy and myogenesis [41, 45]. The results highlight that an acute physiological concentration of H_2_O_2_ increased levels of mmu‐miR‐181a‐5p and mmu‐miR‐378a‐3p, increased mitochondrial content and promoted autophagy in myoblasts. Altogether, the results strongly indicate that changes in mitochondrial content determine myogenesis and this can be manipulated by alterations in the selected exercise‐related miRs.

### Exercise‐related cel‐miR‐77‐5p and cel‐miR‐249‐3p in *C. elegans* display conserved orthologous targets with exercise‐related mmu‐miR‐181a‐5p and mmu‐miR‐378a‐3p

**Fig. 7:**
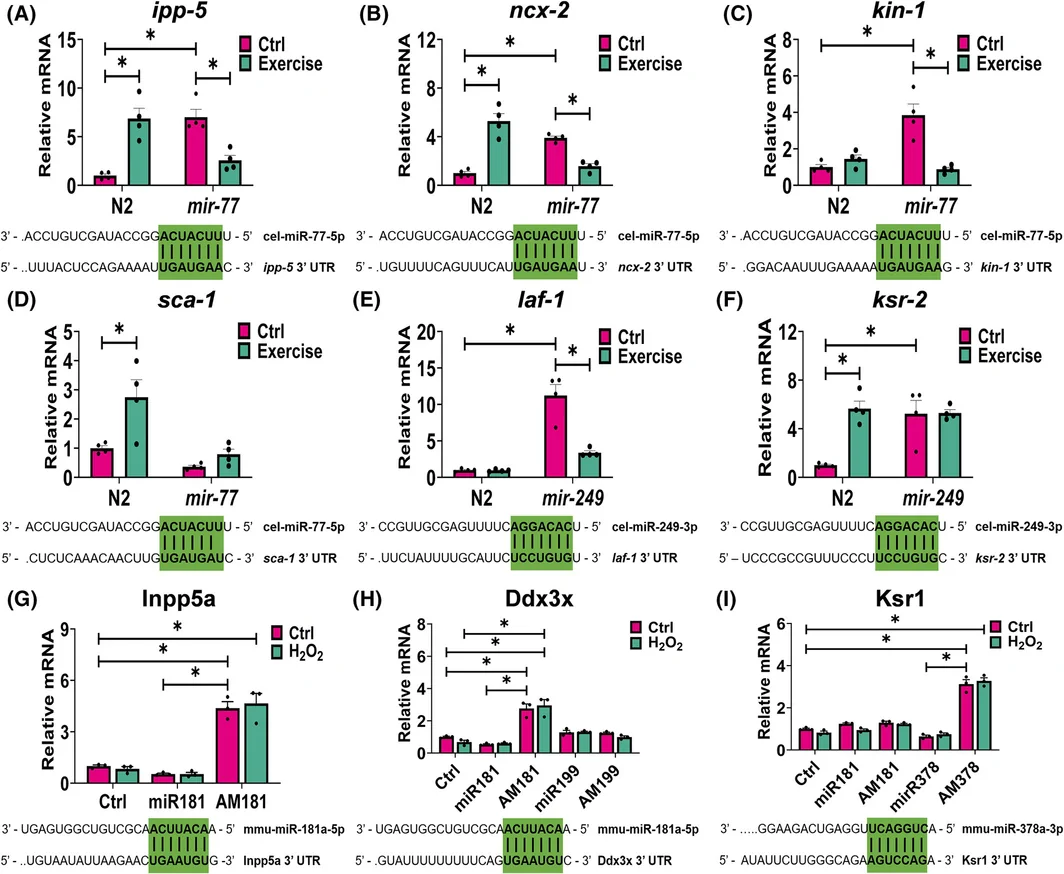
Target genes regulated by the exercise‐related miRs in *C. elegans* and mammalian cells. Expression levels of predicted miRs targets in *C. elegans* including the selected miRs seed sequence and predicted target binding sites: *ipp‐5* (A), *ncx‐2* (B), *kin‐1* (C), *sca‐1* (D), *laf‐1* (E) and *ksr‐2* (F). Expression levels of predicted miR targets in myoblasts including miRs seed sequence and predicted target binding sites: Inpp5a (G), Ddx3x (H) and Ksr1 (I). The green highlighted sequences beneath each graph represent the seed sequence–complementary regions between the miRs and their target genes. Graphs are the normalised relative means ± SEM, experiments were performed with *n* = 4 and *P*‐value of <0.05 was considered as statistically significant *(*P* < 0.05), two‐way ANOVA (A–I) was used for significance between groups. *P*‐values (A: N2 vs *mir‐77* = 0.0004; N2: Ctrl vs Exercise = 0.0005; *mir‐77*: Ctrl vs Exercise = 0.0046. B: N2 vs *mir‐77* = 0.0003; N2: Ctrl vs Exercise <0.0001; *mir‐77*: Ctrl vs Exercise = 0.0023. C: N2 vs *mir‐77* = 0.0003; *mir‐77*: Ctrl vs Exercise = 0.0002. D: N2: Ctrl vs Exercise = 0.01. E: N2 vs *mir‐249* < 0.0001; *mir‐249*: Ctrl vs Exercise <0.0001. F: N2 vs *mir‐249* = 0.0028; N2: Ctrl vs Exercise = 0.0014. G: in control group: ctrl vs AM181 < 0.0001, miR181 vs AM181 < 0.0001; Ctrl: ctrl vs H_2_O_2_: AM181 < 0.0001. H: in control group: ctrl vs AM181 < 0.0001, miR181 vs AM181 < 0.0001; in H_2_O_2_ group: ctrl vs AM181 < 0.0001; Ctrl: ctrl vs H_2_O_2_: AM181 < 0.0001. I: in control group: ctrl vs AM378 < 0.0001, miR378 vs AM378 < 0.0001; Ctrl: ctrl vs H_2_O_2_: AM378 < 0.0001).

Although the exercise‐related miRs identified in *C. elegans* and murine myoblasts are not conserved, we hypothesised that these miRs may share similar targets involved in regulating key mechanisms of muscle function. In a previous study, a proteomics analysis following exercise in the N2 wild‐type strain was performed, identifying 3590 proteins, with 66 upregulated and 67 downregulated proteins compared to nonexercised controls [27]. A comparative analysis between the predicted targets of cel‐miR‐77‐5p and cel‐miR‐249‐3p and the proteomics data was performed. This analysis identified 51 predicted targets for cel‐miR‐77‐5p, including 21 upregulated and 25 downregulated proteins from the dataset, and 19 predicted targets for cel‐miR‐249‐3p, encompassing 8 downregulated and 11 upregulated proteins. Within the predicted targets of cel‐miR‐77‐5p, 10 proteins associated with muscle contraction were identified: NCX‐2, SCA‐1, IPP‐5, KIN‐1, SCPL‐4, LMP‐1, CPZ‐2, UNC‐112, GRD‐5 and T09B4.5. For cel‐miR‐249‐3p, five proteins related to muscle contraction were highlighted: LAF‐1, KSR‐2, UNC‐54, MAX‐2 and PUF‐9. Further analysis using TargetScan (https://www.targetscan.org) identified the top four potential targets of cel‐miR‐77‐5p (*ncx‐2*, *sca‐1*, *ipp‐5*, *kin‐1*) and two potential targets of cel‐miR‐249‐3p (*laf‐1*, *ksr‐2*) (Table 2). Subsequent qPCR analysis in the *C. elegans* N2 wild‐type strain confirmed that following exercise there was an upregulation of genes involved in intracellular Ca^2+^ regulation, *ipp‐5*, *ncx‐2* and *sca‐1* (Fig. 7A,B,D). In the *mir‐77* mutant strain, there was basally elevated levels of *ipp‐5*, *ncx‐2* and *kin‐1* compared to N2 (Fig. 7A–C) suggesting that they are potential targets of cel‐miR‐77. For cel‐miR‐249‐3p, qPCR analysis demonstrated elevated expression levels of *laf‐1* and *ksr‐2* in the *mir‐249* mutant strain compared to N2 (Fig. 7E,F). These results suggest that *ncx‐2*, *kin‐1* and *ipp‐5* are potential targets of cel‐miR‐77‐5p, whereas *laf‐1* and *ksr‐2* are potential targets of cel‐miR‐249‐3p.

**Table 2:** Summary of putative miR targets with known or potential role in muscle and their binding miRs

Targets	Mammalian ortholog	Functions in muscle	Targeting miRs in *C. elegans*	Targeting miRs in mammals	Proteomics		
						Log_2_ (FC)	*P*‐value
*ipp‐5*	Inpp5a	Contributes to Ca^2+^ metabolism [51, 65]	cel‐miR‐77‐5p	mmu‐miR‐181a‐5p [67]	↑ after exercise	0.81	1
*sca‐1*	Atp2a1	Contributes to Ca^2+^ sequestration in ER [63, 64]	cel‐miR‐77‐5p	–	↑ after exercise	0.65	0.003
*ncx‐2*	Slc8a3	Contributes to cellular Ca^2+^ homeostasis [61, 62]	cel‐miR‐77‐5p	–	↑ after exercise	0.54	0.002
*kin‐1*	Prkaca	cAMPK catalytic subunit alpha and can regulate phosphorylation of several Ca^2+^ channels	cel‐miR‐77‐5p	–	↑ after exercise	0.46	0.167
*laf‐1*	Ddx3x	ATP dependent RNA helicase	cel‐miR‐249‐3p	mmu‐miR‐181a‐5p [76] mmu‐miR‐199a‐5p	↓ after exercise	−0.13	0.274
*ksr‐2*	Ksr1	Promotes activation of MAPK1 [52, 78, 79]	cel‐miR‐249‐3p	mmu‐miR‐181a‐5p mmu‐miR‐378a‐3p [77]	–	–	–

While certain miRs are conserved across species [46, 47], many exhibits species‐specific sequences or significant divergence. Our data suggest that mmu‐miR‐181a‐5p, mmu‐miR‐199a‐5p, and mmu‐miR‐378a‐3p in mice share analogous physiological roles with cel‐miR‐77‐5p and cel‐miR‐249‐3p in *C. elegans*, particularly in regulating Ca^2+^ homeostasis and metabolic pathways. To explore potential conserved molecular targets, we utilised WORMHOLE (https://wormhole.jax.org/) [48], to identify orthologous genes targeted by cel‐miR‐77‐5p and cel‐miR‐249‐3p in *C. elegans* and mmu‐miR‐181a‐5p, mmu‐miR‐199a‐5p and mmu‐miR‐378a‐3p in mice, thus revealing putative shared targets between the two species. Our analysis identified that cel‐miR‐77‐5p shared 12 putative targets with mmu‐miR‐181a‐5p, 2 with mmu‐miR‐199a‐5p and 2 with mmu‐miR‐378a‐3p. Similarly, cel‐miR‐249‐3p exhibits 16 shared targets with mmu‐miR‐181a‐5p, 9 with mmu‐miR‐199a‐5p and 2 with mmu‐miR‐378a‐3p. Interestingly, Inpp5a (Inositol Polyphosphate‐5‐Phosphatase A), an ortholog of *ipp‐5*, emerged as a shared target between cel‐miR‐77‐5p and mmu‐miR‐181a‐5p. Ddx3x (DEAD‐box helicase 3 X‐linked), an ortholog of *laf‐1*, was identified as a potential target of cel‐miR‐249‐3p and both mmu‐miR‐181a‐5p and mmu‐miR‐199a‐5p. Ksr1 (Kinase Suppressor of Ras), an ortholog of *ksr‐2*, was a predicted shared target of cel‐miR‐249‐3p and with mmu‐miR‐181a‐5p and mmu‐miR‐378a‐3p. To validate these putative shared targets, qPCR confirmed an increased expression of Inpp5a in the AM181 group, both with and without H_2_O_2_ treatment (Fig. 7G). Additionally, Ddx3x levels were upregulated following mmu‐miR‐181a‐5p inhibition, but no changes were observed with mmu‐miR‐199a‐5p treatment (Fig. 7H). Moreover, Ksr1 levels were elevated under mmu‐miR‐378a‐3p inhibition, with no significant correlation observed with mmu‐miR‐181a‐5p (Fig. 7I). These findings suggest a conserved regulatory mechanism where cel‐miR‐77‐5p and mmu‐miR‐181a‐5p target *ipp‐5*/Inpp5a, cel‐miR‐249‐3p and mmu‐miR‐181a‐5p target *laf‐1*/Ddx3x, cel‐miR‐249‐3p and mmu‐miR‐378a‐3p target *ksr‐2*/Ksr1 (Fig. 7A,E,F,H,I). This comparative approach sheds light on potential evolutionary conserved mechanisms underlying physiological adaptations to exercise, revealing putative conserved molecular targets regulated by exercise‐related miRs across species.

### AM181 pretreatment decreases Ca^2+^ release in myotubes

**Fig. 8:**
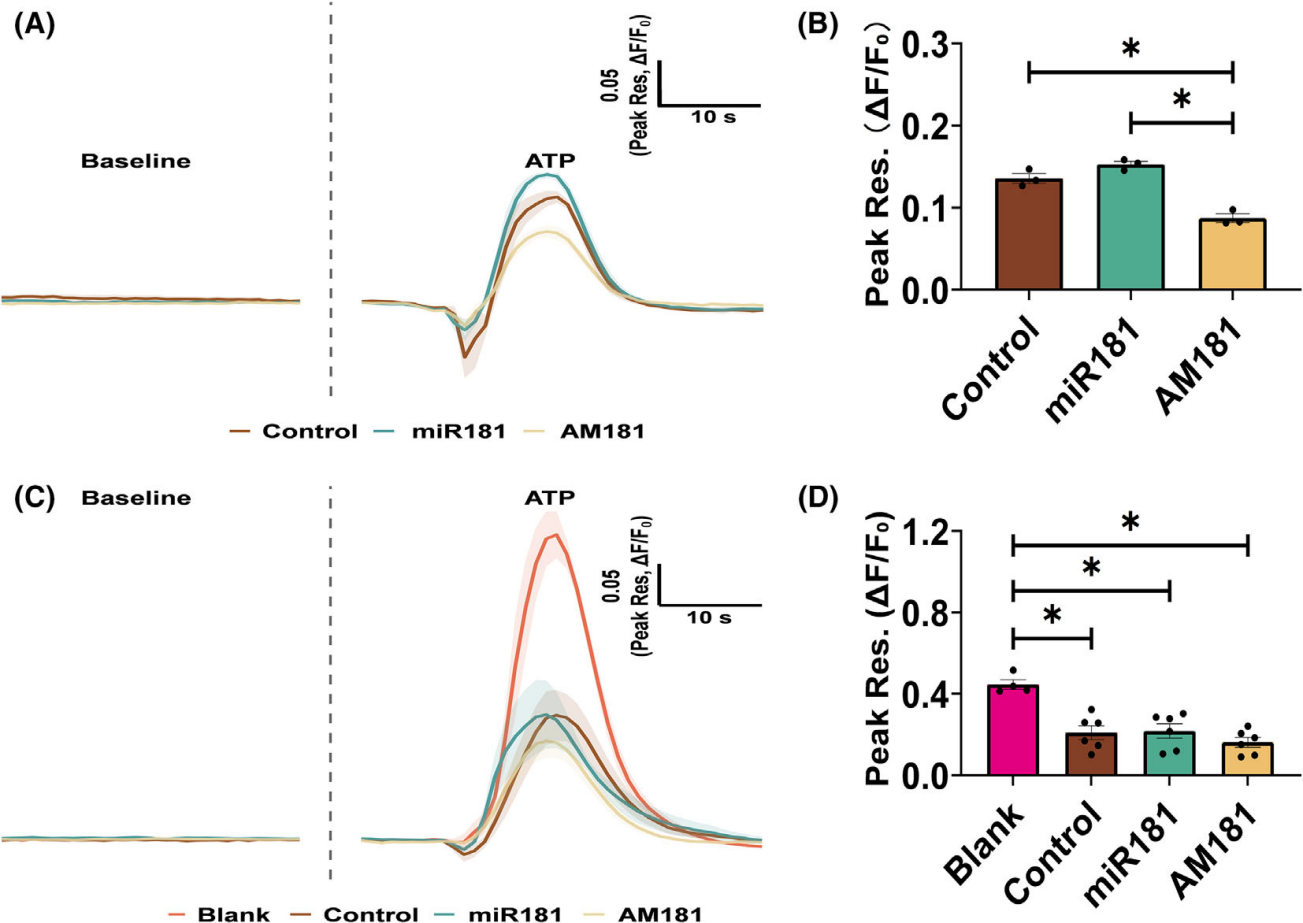
AM181 decreases Ca^2+^ release in myotubes. Ca^2+^ imaging was performed in C2C12 myotubes loaded with Flou‐4AM, that were pretreated with miR181 or AM181. ATP was used to added at indicated timepoint to stimulate Ca^2+^ release both without (A, B) and with Suramin a P2Y2R purinergic receptor inhibitor (C, D). Graphs are the normalised relative means ± SEM and experiments were performed with *n* = 3 (without Suramin), *n* = 6 (with Suramin) and *P*‐value of <0.05 was considered as statistically significant *(*P* < 0.05), one‐way ANOVA (A–D) was used for significance between groups. *P‐*values (B: Ctrl vs AM181 = 0.0012; miR181 vs AM181 = 0.0002. D: Blank vs Ctrl = 0.0005; Blank vs miR181 = 0.0007; Blank vs AM181 < 0.0001).

Ca^2+^ homeostasis and regulation are key during contractile activity and for the adaptive response to exercise. INPP5a is a negative regulator of IP_3_ resulting in decreased Ca^2+^ release from the ER/SR. The Na^+^/Ca^2+^ pump NCX‐3 can also regulate intracellular Ca^2+^ levels and prevent mitochondrial Ca^2+^ overload during conditions of ischaemia and hypoxia. cel‐miR‐77‐5p is predicted to target both *ipp‐5* the *C. elegans* ortholog of Inpp5a and *ncx‐2* the ortholog of the Na^+^/Ca^2+^ exchanger. Following exercise, levels of *ipp‐5* and *ncx‐2* increased and the *mir‐77* mutant strain was characterised by basally elevated levels of *ipp‐5* and *ncx‐2* compared to the N2 strain (Fig. 7A,B). As cel‐miR‐77‐5p levels decreased following exercise (Fig. 1A) and there was a corresponding increase in *ipp‐5* levels, suggesting that *ipp‐5* is a putative target of cel‐miR‐77‐5p (Fig. 7A). Inpp5a is also a predicted target of mmu‐miR‐181a‐5p. In C2C12 myoblasts, miR181 overexpression led to decreased levels of Inpp5a while AM181 treatment resulted in increased levels of Inpp5a (Fig. 7G). To determine the physiological consequences of altered Inpp5a levels, intracellular Ca^2+^ imaging was performed on myotubes that were pretreated with miR181 or AM181. ATP acting via G‐protein coupled P2Y2R results in a rapid transient spike in intracellular Ca^2+^ which was attenuated by suramin, an inhibitor of P2Y2R. Cells pretreated with AM181 demonstrated a significantly decreased Ca^2+^ release profile in response to ATP, while miR181 treatment resulted in a nonsignificant increase in Ca^2+^ release (Fig. 8A,B). Pre‐incubation with suramin, resulted in an anticipated ~50% decrease in Ca^2+^ transients and there was no effect of either miR181 or AM181 pretreatments, indicating that changes observed were likely due to IP_3_ mediated Ca^2+^ signalling (Fig. 8C,D). We hypothesise that in the absence of suramin, AM181 resulted in increased levels of Inpp5a, which decreased IP_3_ activity and was observed as a reduction in Ca^2+^ transients. These results demonstrate that exercise‐related miRs, although not conserved, target physiologically relevant pathways associated with dynamic Ca^2+^ homeostasis and the adaptive response to exercise.

Small RNA sequencing revealed exercise‐induced changes in miRs, increased levels of cel‐miR‐57‐5p and cel‐miR‐249‐3p but decreased levels of cel‐miR‐72‐3p and cel‐miR‐77‐5p. *mir‐57* and *mir‐249* mutant strains exhibited enhanced fertility, survival and lifespan, while *mir‐72* and *mir‐77* mutant strains demonstrated diminished fertility, survival and lifespan. Moreover, both *mir‐77* and *mir‐249* mutant strains exhibited reduced fitness and impaired mitochondrial morphology following exercise. While the seed sequences of the exercise responsive miRs identified in *C. elegans* are not conserved in mammals, literature‐based identification of exercise‐related mammalian miRs, such as mmu‐miR‐181a‐5p, mmu‐miR‐199a‐5p and mmu‐miR‐378a‐3p were confirmed following acute H_2_O_2_ treatment of myoblasts. Increased levels of mmu‐miR‐181a‐5p and mmu‐miR‐378a‐3p enhanced mitochondrial content and myogenesis. Comparative analysis of predicted targets identified shared pathways, particularly in regulating Ca^2+^ homeostasis, with both mmu‐miR‐181a‐5p and cel‐miR‐77‐5p targeting Inpp5a/*ipp‐5* to modulate Ca^2+^ metabolism. Ca^2+^ imaging in myotubes demonstrated decreased Ca^2+^ flux following AM181 treatment that was accompanied by increased Inpp5a levels, potentially decreasing IP_3_ mediated Ca^2+^ release. Additionally, mmu‐miR‐378a‐3p and cel‐miR‐249‐3p targeted Ksr1/*ksr‐2*, involved in activating the MAPK pathway to regulate mitochondrial dynamics. These findings suggest conserved regulatory mechanisms linking miRs to Ca^2+^ homeostasis and mitochondrial function. Together, the data indicate common pathways respond to exercise in *C. elegans* and mammalian cells that are regulated by divergent miRs.

## Discussion

Regular exercise helps maintain muscle mass and ameliorate age‐related muscle atrophy. Exercise enhances mitochondrial biogenesis and the adaptive response to exercise results in the activation of redox‐sensitive transcription factors influencing gene expression [49]. However, the intricate mechanisms underlying the adaptive response to an acute physiological stress generated by exercise remain incompletely understood. Previous research has demonstrated that exercise enhances mitochondrial function, stress resistance and longevity in *C. elegans* [25, 27]. miRs have been identified as central regulators of post‐transcriptional control in muscle proliferation, myogenesis and homeostasis [41, 44, 50]. In this study, we identified common pathways regulated by exercise‐related miRs. In *C. elegans*, a 5‐day chronic exercise regimen enhanced mitochondrial content, survival, lifespan, and fitness [27]. Following exercise, cel‐miR‐57‐5p and cel‐miR‐249‐3p levels increased, while cel‐miR‐72‐3p and cel‐miR‐77‐5p levels decreased. Phenotypic analyses of *mir‐57* and *mir‐249* mutant strains revealed reduced fertility but increased survival and extended lifespan. Conversely, *mir‐72* and *mir‐77* mutants exhibited heightened fertility, decreased survival and shortened lifespan. In myoblasts, mmu‐miR‐181a‐5p and mmu‐miR‐378a‐3p enhanced mitochondrial capacity and myogenesis, whereas mmu‐miR‐199a‐5p overexpression resulted in disrupted mitochondria and impaired myogenesis. Computational analyses identified that both mmu‐miR‐181a‐5p and cel‐miR‐77‐5p target Inpp5a/*ipp‐5*, are involved in regulating Ca^2+^ metabolism [51]. Furthermore, mmu‐miR‐378a‐3p and cel‐miR‐249‐3p were found to target Ksr1/*ksr‐2*, activating the MAPK pathway that can regulate mitochondrial biogenesis [52].

The hormesis effect of exercise‐induced ROS has been widely documented [53, 54]. A short bolus of H_2_O_2_ to myoblasts induced beneficial adaptive responses, including mitochondrial biogenesis and myogenesis [27]. Conversely, chronically elevated ROS levels can be deleterious, causing oxidative damage and impairing mitochondrial function [53]. Previously it was observed that acute H_2_O_2_ treatment induces the nuclear localisation of redox‐sensitive transcription factors such as NRF2, NFκB and FOXO3, all of which have been associated with mitochondrial biogenesis in response to exercise [27]. Additionally, many of the exercise‐specific miRs identified from the literature contain binding sites for these redox‐regulated transcription factors (Table 1). Previous studies have established the regulatory role of miR‐181 in mitochondrial homeostasis [55] and identified mmu‐miR‐181a‐5p as a regulator of mitochondrial dynamics, targeting p62/SQSTM1, Protein DJ‐1 and Parkin in skeletal muscle [41]. Additionally, miR‐199‐5p has been linked to smooth muscle hypertrophy [56], with elevated levels in human dystrophic muscle and associated with impaired myofibres in zebrafish [43]. Recent research has also associated elevated miR‐199‐5p with cardiovascular dysfunction and reduced exercise endurance at high altitudes [57]. These findings suggest that miR‐199‐5p may serve as a negative regulator of muscle development, consistent with our findings. miR‐378 has been reported to regulate glucose tolerance [58], mitochondrial homeostasis [59] and muscle development [60]. Furthermore, miR‐378 has been identified as a promoter of autophagy in skeletal muscle [45], supporting the data obtained here. Unexpectedly, following H_2_O_2_ treatment, the miR378 group demonstrated decreased mitochondrial content, increased mitochondrial ROS and impaired myogenesis, indicating a shift from the anticipated positive effects of mmu‐miR‐378a‐3p to deleterious outcomes in myoblasts following H_2_O_2_ treatment. A similar phenomenon was observed in *C. elegans*, particularly with cel‐miR‐249‐3p, where its absence resulted in extended lifespan and survival, but increased mitochondrial fragmentation, decreased stress resistance and longevity following chronic exercise.

Small RNA sequencing identified miRs affected by exercise in N2 and *prdx‐2* mutant strains. Exercise in the N2 strain led to increased levels of 5 miRs, whereas the *prdx‐2* mutant had increased levels of 14 miRs. The small RNA sequencing indicated that cel‐miR‐77‐5p exhibited the most pronounced decrease in both N2 and *prdx‐2* mutants following exercise. To identify potential targets of cel‐miR‐77‐5p, we compared its predicted targets via TargetScan with the upregulated proteins identified from a previous proteomics study [27]. A number of potential targets were identified including, *ncx‐2*, the ortholog of solute carrier family 8 member A3 (Slc8a3), that plays a crucial role in cellular Ca^2+^ homeostasis in muscle [61, 62]; *sca‐1*, ortholog of ATPase sarcoplasmic/endoplasmic reticulum Ca^2+^ transporting 1 (Atp2a1), involved in Ca^2+^ sequestration during excitation–contraction coupling [63, 64]; Additionally, *ipp‐5*, the ortholog of Inpp5a, plays a significant role in muscle function by hydrolysing IP_3_ to regulate Ca^2+^ signalling [65, 66].

Inpp5a was previously identified as a predicted target of mmu‐miR‐181 in HeLa cells [67] and our results indicate that its ortholog in *C. elegans*, *ipp‐5*, is a putative target of cel‐miR‐77‐5p. Inpp5a encodes the inositol polyphosphate‐5‐phosphatase enzyme, critical for Ca^2+^ signalling, membrane trafficking and cell proliferation in skeletal muscle [68, 69, 70]. Comparing the predicted targets of cel‐miR‐249‐3p with previous quantitative proteomics data [27], four predicted genes are associated with mitochondrial function in skeletal muscle. These include *laf‐1*, the ortholog of Ddx3x, an ATP‐dependent RNA helicase which is implicated in mitochondrial DNA depletion and DDX3X syndrome [71], *unc‐54*, the ortholog of Myh7, a motor protein with ATPase activity essential for muscle contraction [72, 73], *max‐2*, the ortholog of Pak1, required for skeletal muscle mitochondrial function [74]; and *puf‐9*, the ortholog of Pum2, which regulates mitochondrial dynamics and mitophagy [75]. Among these, Ddx3x/*laf‐1* emerges as a shared target of both mmu‐miR‐181 and mmu‐miR‐199 [76], our data confirmed Ddx3x as a putative target of mmu‐miR‐181a‐5p in myoblasts. Additionally, Ksr1 is a shared predicted target of both mmu‐miR‐181a‐5p and mmu‐miR‐378a‐3p, as identified via TargetScan. qPCR results confirmed Ksr1 as a putative target of mmu‐miR‐378a‐3p, aligning with previous findings that identified Ksr1 as a target of miR‐378 in rat cardiomyocytes [77]. Ksr1 is a scaffold protein integral to the regulation of the MAPK signalling pathway, which plays a pivotal role in the adaptive response to exercise, encompassing critical aspects such as mitochondrial capacity and muscle function [78, 79].

16 miRs have been suggested as muscle‐specific in the body wall muscle of *C. elegans* [80]. These include miR‐241, miR‐230, miR‐77, miR‐5551, miR‐34, miR‐228, miR‐let‐7, miR‐80, miR‐250, miR‐239a, miR‐239b, miR‐392, miR‐270, miR‐4937, miR‐793, and miR‐799 [80]. Following chronic exercise, three of these miRs (cel‐miR‐77‐5p, miR‐80 and miR‐250) were downregulated in both N2 and *prdx‐2* mutant strains. Interestingly, in the absence of PRDX‐2, there was an increased level of miR‐34, a muscle‐specific miR and an ortholog of vertebrate miR‐34. miR‐34 overexpression has been shown to induce cellular senescence [81], suppress muscle development [82] and impairment of the insulin signalling pathway [83], mirroring similar phenotypes observed in the *prdx‐2* mutant [27, 84, 85]. It is worth mentioning that three miRs (miR‐37, miR‐39 and miR‐72) exhibited divergent changes in levels following exercise: decreased in the N2 wild‐type strain but increased in the *prdx‐2* mutant strain. Interestingly, arm changes in these miRs were evident in the *prdx‐2* mutant; miR‐37‐5p switched to miR‐37‐3p, miR‐39‐5p switched to miR‐39‐3p and miR‐72‐3p switched to miR‐72‐5p, although the overall levels of the passenger strand were still less than the levels of its guide strand. Arm switching is a remarkably dynamic process that can vary across different tissues, developmental stages or cellular conditions [86]. However, the detailed molecular mechanisms underlying arm switching are not fully understood [87]. In the context of our experimental findings, two potential factors may contribute to the observed phenomenon. First, acute oxidative stress induced by exercise and the absence of PRDX‐2 could contribute, as oxidative stress is known to regulate RNA processing and the activity of key enzymes involved in miR assembly [88]. Second, our analysis of prior proteomics data suggests a possible role for PRDX‐2 in miR biogenesis [27]. However, these hypotheses require further investigation to elucidate the precise molecular mechanisms underlying the observed arm switching.

Exercise can dramatically change mitochondrial morphology and it has been demonstrated in *C. elegans* to initially induce mitochondrial fragmentation followed by mitochondrial fusion after a recovery period, a cycle that becomes disrupted during ageing [25, 28]. Interestingly, in contrast to the beneficial effects in the wild‐type N2 strain, long lived *C. elegans* mutant strains such as *daf‐2*, *isp‐1*, *nuo‐6* and *eat‐2* had reduced physical fitness and longer recovery periods following a swimming exercise protocol [25]. Exercise can also modulate interorganelle communication between mitochondria and other organelles including lysosomes, lipid stores and the ER/SR [28, 89]. Interorganelle crosstalk and the exchange of material and information in response to biological perturbations is crucial in order to respond to changes in cellular homeostasis [90]. Communication between the ER and mitochondria at contact sites is required for the transfer of Ca^2+^, lipids and metabolites [91]. MERCS are dynamic structures that can respond to changes in cellular homeostasis and help determine sites of mitochondrial fission and fusion ultimately regulating mitochondrial morphology but also Ca^2+^ and lipid homeostasis [90, 92, 93]. Ca^2+^ is essential for muscle contraction and mitochondrial metabolism, which are fundamental during exercise [94]. Regular exercise enhances skeletal muscle Ca^2+^ handling and improves running performance in mice [95]. Impaired Ca^2+^ homeostasis can lead to an imbalance in Ca^2+^ uptake, changes in mitochondrial membrane potential and shifts in fibre type, all of which collectively influence exercise capacity in mice [96]. Conversely, excessive mitochondrial Ca^2+^ accumulation is associated with the induction of apoptotic signalling and muscle dystrophy [97]. Thus, maintaining Ca^2+^ balance is crucial for optimal muscle function and development. Our findings indicate that the exercise‐induced reduction in cel‐miR‐77‐5p levels likely represents an acute adaptive response to enhance the expression of its target genes (*ipp‐5*, *ncx‐2* and *kin‐1*), regulate Ca^2+^ homeostasis and mitochondrial function. In contrast, the complete absence of cel‐miR‐77‐5p in the *mir‐77* mutant may disrupt the regulation of these targets or lead to their uncontrolled upregulation, resulting in compromised Ca^2+^ handling, apoptotic signalling and mitochondrial dysfunction. This dysregulation could contribute to decreased mitochondrial potential and reduced lifespan observed in the *mir‐77* mutant following exercise. Therefore, while the downregulation of cel‐miR‐77‐5p during exercise appears to facilitate adaptive responses, its absence in the *mir‐77* mutant emphasises the critical role of cel‐miR‐77‐5p in maintaining Ca^2+^ homeostasis and mitochondrial health over the long term.

MAPK represents a family of serine/threonine kinases that are critical components of evolutionarily conserved signalling pathways. In skeletal muscle, MAPK is indispensable for processes such as muscle differentiation, regeneration and adaptations to physiological stress [98]. Exercise‐induced MAPK activation stimulates the mitochondrial regulator PGC‐1α, thereby promoting mitochondrial biogenesis and facilitating muscle adaptation [99]. Additionally, Ddx3x has been implicated in mitochondrial DNA depletion in skeletal muscle, further underscoring the importance of these pathways in mitochondrial health [71]. Given that both *ksr‐2* (the ortholog of Ksr1, which activates MAPK) and *laf‐1* (the ortholog of Ddx3x) are targets of cel‐miR‐249‐3p, the observed increase in cel‐miR‐249‐3p following exercise appears paradoxical when considering the *mir‐249* mutant exhibits increased mitochondrial membrane potential and extended lifespan. This contradiction suggests that the exercise‐induced upregulation of cel‐miR‐249‐3p might be an adaptive response aimed at fine‐tuning mitochondrial capacity to maintain a balance in mitochondrial function. In contrast, the enhanced mitochondrial membrane potential and extended lifespan observed in the *mir‐249* mutant indicate that the absence of cel‐miR‐249‐3p leads to the upregulation or increased activity of its targets, including *ksr‐2* and *laf‐1*, potentially resulting in alterations in mitochondrial dynamics. Therefore, while cel‐miR‐249‐3p may serve to modulate these processes under normal conditions, its absence in the *mir‐249* mutant might disrupt this regulation, leading to more pronounced effects on mitochondrial capacity and longevity. In the future, muscle‐specific miR sequencing and Ca^2+^ imaging in *C. elegans* would provide a more direct and comprehensive approach to understand the underlying mechanisms involved in the adaptive response to exercise.

Furthermore, putative conserved gene targets, such as Inpp5a/*ipp‐5* and Ksr1/*ksr‐2*, of exercise‐sensitive miRs from mammals and *C. elegans* have been suggested in this work, but mechanistic assays such as luciferase reporter assays are required to confirm a direct interaction between the miRs and their predicted targets. Alternatively, using specific pathway inhibitors of MAPK/ERK signalling or autophagy in combination with miR‐resistant mRNA constructs would clarify if the effects observed in the models are direct or secondary effects due to changes in redox or differentiation states. Similarly, it was not possible to determine the tissue‐specific roles of the miRs when using the *C. elegans* mutant strains. As these mutant strains have lost expression of these miRs, it may result in compensatory mechanisms that occur during early development and mask their true biological function.

In summary, the results highlight the involvement of cel‐miR‐57‐5p, cel‐miR‐72‐3p, cel‐miR‐77‐5p and cel‐miR‐249‐3p in the regulation of fertility, survival and mitochondrial capacity in *C. elegans*. Similarly, mmu‐miR‐181a‐5p, mmu‐miR‐199a‐5p and mmu‐miR‐378a‐3p play key regulatory roles on mitochondrial content and myogenesis in C2C12 myoblasts. Computational analysis and qPCR validation identified Inpp5a/*ipp‐5* as a putative shared target of mmu‐miR‐181a‐5p and cel‐miR‐77‐5p, associated with Ca^2+^ homeostasis in skeletal muscle. Additionally, Ksr1/*ksr‐2* was identified as a potential shared target of mmu‐miR‐378a‐3p and cel‐miR‐249‐3p, which is linked to MAPK activation for the regulation of mitochondrial biogenesis. Collectively, this data underscores the critical roles of miRs and suggests conserved regulatory mechanisms governing mitochondrial function across species.

## Methods

### *Caenorhabditis elegans* strains and swimming exercise

*Caenorhabditis elegans* were cultured on nematode growth medium (NGM) plates seeded with *E. coli* (OP50) at 20 °C. *Caenorhabditis elegans* stains N2 wild‐type, MT16311 (*mir‐77(n4286) II*), MT13015 (*mir‐72(n4130) II*), MT16848 (*mir‐249(n4983) X*), VC347 (*mir‐57(gk175) II*) and SJ4103 (*zcIs14 [myo‐3::gfp(mit)]*) strains were obtained from the Caenorhabditis Genetics Center (CGC, University of Minnesota, USA). The outcrossing of *mir‐77* and *mir‐249* strains with mitochondrial muscle GFP reporter SJ4103 *zcIs14 [myo‐3::GFP(mit)]* were performed in this project. The 5‐day swimming protocol was performed as previously described [27].

### C2C12 cell culture

C2C12 myoblasts (obtained from ATCC, RRID:CVCL_0188) were cultured in 10% growth medium supplemented with DMEM, 10% fetal bovine serum and 1% penicillin/streptomycin. Cells were maintained under standard conditions at 37 °C with 5% CO_2_. Authentication included verification of myogenic potential through a differentiation protocol and visualisation of myotubes and morphological analyses. Mycoplasma tests were performed using a qPCR approach and visualisation of cultures following DAPI staining [100]. To investigate the impact of cholesterol‐linked miR‐181, 199, 378 mimic and antagomiR‐181, 199, 378, C2C12 myoblasts at 50–60% confluency were subjected to treatment with a final concentration of 100 nm for 24 h prior to an acute 10‐min exposure to 25 μm H_2_O_2_, that has previously been demonstrated to improve myogenesis and increase mitochondrial content [27]. Following treatment, media were exchanged, and cells were allowed to recover for 24 h before protein and RNA were extracted. In order to assess the impact of selected miRs on myotube formation, C2C12 cells at 70–80% confluence were treated with miRs or antagomiRs (100 nm) for 24 h prior to H_2_O_2_ treatment, growth media were replaced with differentiation media and the cells were cultured in differentiation media for a period of 5–7 days. MF20 staining was performed to determine effects on myogenesis.

### Western blotting

Proteins were quantified using the Bradford assay, 20 μg of protein were separated on 12% SDS/PAGE gels and transferred using a semi‐dry blotter. After blocking with 5% milk, the membranes were incubated overnight with primary antibodies including rabbit anti‐TOM20 (#42406; CST, Massachusetts, USA), VDAC1 (#ab14734; Abcam, Cambridge, UK), SIRT1 (#ab110304; Abcam), ATG5 (#ab108327; Abcam), ULK1 (#ab128859; Abcam), SQSTM1/p62 (#88588; CST) and LC3b (#ab192890; Abcam) at a 1:1000 dilution. Following washing with TBS‐T, the membranes were incubated with secondary IRDye 800CW Goat anti‐Mouse IgG antibodies (#925–32 210; LI‐COR Biosciences; Lincoln, NE, USA) at a 1:10000 dilution in the dark for 1 h. The acquired images of the blot were captured using the Odyssey Fc imaging system. Ponceau S staining (#A40000279; Thermo Fisher Scientific; Waltham, MA, USA) was used to confirm total protein loading for normalisation. The quantification and normalisation of the blots were analysed utilising Image Studio Lite.

### PCR for genotyping

DNA extraction from *C. elegans* was performed using worm lysis mix (WLM) containing proteinase K (#03115828001; MERCK, Millipore, USA). About 15 worms were added to a PCR tube along with 15 μL of WLM and placed at −80 °C for 1 h. Subsequently, samples were transferred to a thermocycler and subjected to incubation at 65 °C for 1 h, followed by a denaturation step at 95 °C for 5 min and then cooled on ice. The PCR reaction mix was prepared by combining 12.5 μL of MyTaq™ Reaction Buffer Red (#R2523; MERCK), 0.5 μL each of forward and reverse primers, 9 μL of nuclear‐free water, and 2.5 μL of DNA. The amplification process commenced with an initial denaturation step at 95 °C for 10 min, followed by 40 cycles consisting of denaturation at 95 °C for 15 s, annealing at 56 °C for 15 s, and extension at 72 °C for 90 s. Finally, the reaction was held at 4 °C. Subsequently, the PCR products were subjected to agarose electrophoresis, and gel images were captured using the LI‐COR Odyssey. Sequences of primers used for genotyping strains are listed in Table 3.

**Table 3:** Primers used in this study.

*Caenorhabditis elegans*	
CDC‐42	Forward: AGCCATTCTGGCCGCTCTCG Reverse: GCAACCGCTTCTCGTTTGGC
PMP‐3	Forward: CGGTGTTAAAACTCACTGGAGA Reverse: TCGTGAAGTTCCATAACACGA
*ipp‐5*	Forward: ACTGGAGATCATAAGCCGGTC Reverse: GAGTTCGCGCAAGCCAATTT
*kin‐1*	Forward: GTGGTTGTAGAGAGCCAGGT Reverse: CACTTGCTTGAGTTTTACAACCTTT
*ncx‐2*	Forward: GTGCATCATCGTCTTGGTGC Reverse: ACATTGCTGTCATCCACCTCA
*sca‐1*	Forward: CCACCATCTTCCAGATCACCC Reverse: CTCTGATTGAGGGAGCACTGT
*ksr‐2*	Forward: GATGAAAAGAAGAAAAAGCGCGGA Reverse: AGGCGGTGGTGCATTGATAG
*laf‐1*	Forward: GCGTGGAGCTTCTGATTTGG Reverse: TGGAGCTGTTCCGGTTCTAAA
*C. elegans* genotyping	
cel‐miR‐57‐5p	OF: CCCGACAAATCCTCAAAG OR: GAAGCCAGGCTCAAATCTCTACAG IR: GAGCTAGACTACAAGGTGCAC
cel‐miR‐72‐3p	OF: CCGAATTCCAGCTAGGTTTC OR: CAAATACTCGTGCTGCAACG IR: CTTTAGCGGCGAAGAGTTAAGAGG
cel‐miR‐77‐5p	OF: GACATAAACGGCGGCAAAG OR: GATATCGGTGCCGTCTTCTTTG IR: CAAACGCCACAAAGAAACCCC
cel‐miR‐249‐3p	OF: GGGGTGCAAGACTACAATAC OR: CAGAGACAAACGGAATAGCGC IR: GTGAGAATGTGTAGAGACGCAG
C2C12	
S29	Forward: ATGGGTCACCAGCAGCTCTA Reverse: GTATTTGCGGATCAGACCGT
Ddx3x	Forward: TGAGAGGGCCTTTGCGG Reverse: GATATAACGCCCTTTGCTTGC
Inpp5a	Forward: GGGTCAGATGGGTCTCATTTCC Reverse: TGTGTGTGCAGGACCTGGTAAA
Ksr1	Forward: GCCCTGACAAAGAAGGAGCA Reverse: TGTCTGGGAAGCTGAACCTG

### qPCR

RNA extraction from both cellular and *C. elegans* samples was performed using TRIzol (#15596018; Life Technologies, Waltham, MA, USA) [101]. For mRNA synthesis, 500 ng of RNA was mixed with 1 μL of random hexamers and incubated at 65 °C for 10 min. This was combined with a master mix including 4 μL of RT buffer, 2 μL of DTT (#Y00147; MERCK), 1 μL of dNTP, 1 μL of Superscript II (#100004925; MERCK), and 1 μL of Ribolock (#EO0381; Thermo Fisher Scientific), followed by incubation at 42 °C for 60 min. For miR synthesis, a mixture was prepared by combining 2 μL of 5× miRCury RT reaction buffer, 4.5 μL of RNase‐free water, 1 μL of miRCury RT Enzyme mix, 0.5 μL of synthetic RNA spike in and 2 μL of 5 ng RNA and incubated at 42 °C for 60 min, followed by a final incubation at 95 °C for 5 min. Subsequent qPCR analysis was conducted using the SYBR Green Master Mix (#339347; Qiagen, Venlo, the Netherlands) in a 10 μL reaction volume. S29 was used for normalisation of mRNA in cells and miRs were normalised to unisp6 or U6; RNA from worms was normalised to CDC42, both were calculated using the delta–delta Ct method. The sequences of primers used in this study are listed in Table 3.

### microRNA sequencing in *C. elegans*

RNA isolation from *C. elegans* was performed using TRIzol reagent [101]. Briefly, worms from N2 and *prdx‐2* mutant strains with or without exercise were dislodged from plates and after the removal of bacteria, TRIzol was employed to disrupt the cuticle of the worms. Subsequently, chloroform was added, followed by centrifugation to isolate the aqueous phase. The final steps involved the addition of isopropanol and ethanol, followed by thorough washing to obtain high‐purity RNA. Subsequently, the RNA samples were analysed for small RNA sequencing by TamiRNA (TAmiRNA GmbH Leberstrasse 201 110 Vienna, Austria) and the assessment of next‐generation sequencing (NGS) data quality was conducted through automated and manual evaluation using fastqc v0.11.9 [102] and multiqc v1.10 [103]. Reads from samples underwent adapter trimming and quality filtering with cutadapt v3.3 [104], ensuring a minimum length of 17 nucleotides. Mapping procedures were executed using bowtie v1.3.0 [105] and mirdeep2 v2.0.1.2 [106]. Initially, reads were mapped against the genomic reference WBcel.235 from Ensembl [107], allowing for two mismatches. Subsequently, mapping against miRBase v22.1 was performed [108], filtering for cel‐specific miRs and allowing for one mismatch. Statistical analysis of preprocessed NGS data utilised R v4.0 and packages including heatmap vNA, pca Methods v1.82 and genefilter v1.72. Differential expression analysis was carried out using edger v3.32 [109], employing quasi‐likelihood negative binomial generalised log‐linear model functions. To optimise the false discovery rate (FDR) correction, the independent filtering method of DESeq2 was adapted within edger [110], specifically for the removal of low‐abundance miRs.

### Lifespan assay

The lifespan assay was conducted on adult D6 worms following the completion of the chronic exercise regimen, which was performed twice daily for a total of 5 days [27]. A total of 110–120 worms were carefully transferred to a new plate every 2 days until all worms died. Survival was assessed daily until the last surviving worm exhibited no response.

### Brood size

For the brood size assay, 12 worms at L4 stage worms were transferred to new plates daily. The counting of eggs laid was conducted every 24 h until the cessation of egg‐laying. To determine the egg‐laying rate, worms were allowed to lay eggs over a 5‐h period [111].

### Oxidative stress assays

In the paraquat (#856177; MERCK) and arsenite (#S7400; MERCK) stress assay, 60 worms, postchronic exercise, were picked in individual wells of a 96‐well plate, each well containing either 100 mm paraquat or 2.5 mm arsenite [112]. Worm death was ascertained by the lack of response to a gentle tap with a picker, and survival was assessed every 1–2 h.

### *Caenorhabditis elegans* microscopy

For the MitoTracker (#M7512; Thermo Fisher Scientific) staining in *C. elegans*, 40–45 worms were incubated with 2.5 μm MitoTracker Red for 10 min in the dark one day after the chronic exercise. Following incubation, worms were then transferred to new NGM plates to remove excess staining in the gut. Subsequently, the worms were immobilised using 20 mm levamisole and imaged at 10× magnification. The fluorescence of the entire body and body length of each worm was measured by imagej [27].

To assess mitochondria in the body wall muscle, the SJ4103 strain (*zcIs14 [myo‐3::gfp(mit)]*) was utilised and exposed to chronic swimming. After the exercise, worms were immobilised on an agar slide covered with a coverslip. Subsequently, imaging of mitochondria in the body wall muscles, specifically the area between the pharynx and vulva, was performed at 60× magnification. Quantification was performed on 120–150 images and the analysis involved categorising them into five classes based on the criteria outlined in [26].

### C2C12 immunostaining

For MitoTracker Green (#M7514; Thermo Fisher Scientific) and MitoSOX (#M36008; Thermo Fisher Scientific) staining in cells, medium was removed and washed with Hank's Balanced Salt Solution (HBSS), followed by incubation with a 200 nm MitoTracker Green solution for 30 min. The cells were incubated with a 5 μm MitoSOX Red solution for 10 min. After each incubation step, the cells were washed with HBSS. Following staining, images were captured at 60× magnification. The identification of mitochondrial areas within each cell from various fields of view was based on the presence of MitoTracker Green. Subsequently, the fluorescence intensity within Regions of Interest (ROIs) containing either MitoTracker Green or MitoSOX staining was quantified using ImageJ software as previously described [27].

For MF20 (Developmental studies hybridoma bank; Iowa City, IA, USA) staining of myotubes, media was removed and fixed in ice cold methanol for 5 min, then blocked in 10% horse serum for 1 h at room temperature on rocker, followed by incubation in MF20 primary antibody for overnight. On the subsequent day, the primary antibody was removed and incubated with second antibody for 1 h, followed by 5 min of DAPI staining and the images were captured.

### CeleST

To evaluate the physiological activity of *C. elegans*, CeleST analysis was performed the following methodology outlined in prior research [31]. Briefly, 4–5 worms (with a total of at least 30 worms) were recorded for 30 s using a Nikon LV‐TV microscope set at 1× magnification and the physiological activity quantified using CeleST [31].

### miR target prediction and validation

The predicted targets of the selected miRs were compared to see if common pathways were targeted by exercise‐related miRs in *C. elegans* and mammals. The predicted targets of exercise‐related miRs, cel‐miR‐77‐5p and cel‐miR‐249‐3p in *C. elegans*, mmu‐miR‐181a‐5p, mmu‐miR‐199a‐5p and mmu‐miR‐378a‐3p in mammalian cells, were determined using TargetScan Release 7.1 (https://www.targetscan.org/mmu_71/). In *C. elegans*, there were 145 predicted targets of cel‐miR‐77‐5p, 103 predicted targets of cel‐miR‐249‐3p. In C2C12, there were 1371 predicted targets of mmu‐miR‐181a‐5p, 634 predicted targets of mmu‐miR‐199a‐5p, and 223 predicted targets of mmu‐miR‐378a‐3p. Bioinformatic analysis identified potentially conserved targets of cel‐miR‐77‐5p and cel‐miR‐249‐3p in *C. elegans*, with mmu‐miR‐181a‐5p, mmu‐miR‐199a‐5p and mmu‐miR‐378a‐3p from mice. qPCR was employed to validate a subset of these putative shared targets across the two species. TransmiR v2.0 database (https://www.cuilab.cn/transmir) was used to determine miRs regulated by exercise‐related TFs (NRF2, NF‐κB, FOXO3, STAT3).

### Mitochondrial respiration rates in myoblasts

C2C12 myoblasts were seeded at a density of 8000 cells per well in XF HS Mini Analyzer (Agilent Technologies, Santa Clara, CA, USA) plates, followed by incubation at 37 °C with 5% CO_2_ for 24 h. Cells were then treated with specific miRs and antagomiRs for 24 h. Subsequently, cells were washed once with XF Real‐Time Mito Stress Assay Medium (Agilent Technologies), then replenished with fresh assay medium and incubated for 1 h at 37 °C in a non‐CO_2_ environment. Prior to the assay, the sensor cartridge was hydrated and loaded with mitochondrial stress test compounds: 2 μm oligomycin (Agilent Technologies), 2 μm FCCP (Agilent Technologies) and 1 μm antimycin A (Agilent Technologies). The cartridge was then aligned with the cell plate and calibration was performed using the XF HS Mini Analyzer.

### Ca^2+^ imaging in myotubes

Myotubes growing on glass coverslips were loaded with Fluo‐4AM (2.5 μm) in complete medium for 15 min at 37 °C, followed by a 15‐min wash period in dye‐free complete medium. Coverslips were transferred to a recording chamber filled with physiological buffer (135 mm NaCl, 5 mm KCl, 1 mm MgCl_2_, 2 mm CaCl_2_, 10 mm HEPES, 10 mm glucose, pH 7.45) and imaged using a ZEISS Axiovert 200 M inverted microscope equipped with a xenon lamp (LAMBDA LS, excitation range: 330–650 nm) and Smart Shutter Lambda 10/3 controller. Fluorescence images were captured at 1 Hz for 5 min. Excitation and emission wavelengths were set to 488 nm and 510 nm (long‐pass filter), respectively. Baseline fluorescence was recorded for 1–2 min. At 2 min, ATP (100 μm final concentration) was added, followed by KCl (50 mm final concentration) at 4 min. For inhibition experiments, cells were pre‐incubated with the purinergic receptor antagonist suramin (200 μm) for 2 min prior to recording. Image stacks were analysed using the SARFIA plugin [113] in Igor Pro 8. Regions of interest (ROIs) were defined based on predefined fluorescence thresholds. Data were normalised using the formula ΔF/F0, where F0 represents the average baseline fluorescence over the initial 10 s. Peak responses, mean, area under curve and full‐width half‐maximum (FWHM) fluorescence values were extracted using the NeuroMatic plugin in Igor Pro 8 [114].

### Statistics

Images were analysed semi‐automatically using ImageJ and Image Studio Lite, with subsequent manual corrections. Western blot band intensities were normalised to protein levels determined by Ponceau S staining. For C2C12 microscopy, 3–6 images per biological replicate were randomly captured at 10× or 40× magnification from different fields of view. Myogenic differentiation was assessed via MF20 immunostaining, measuring myotube diameter as the average width of all myotubes per field of view, myotube area as the average area fraction and fusion index as the percentage of nuclear within myotubes relative to the total nuclear. For MitoTracker and MitoSOX, staining was quantified based on relative fluorescence intensity per cell. For SJ4103 strain staining, 130–150 images were analysed in the region between the pharynx and vulva, classified into five predefined categories [25]. Statistical analyses are detailed in the respective figure legends. Differences between two groups were assessed using a Student *t*‐test, while one‐way or two‐way ANOVA was applied for comparisons involving multiple groups. The log‐rank test was used for oxidative stress tolerance assays, and chi‐square tests were performed for categorical distributions. A *P*‐value <0.05 was considered statistically significant. GraphPad Prism 7 was used for all statistical analyses and graphical representations. The source data supporting figure generation are available in the corresponding source data file.

## Ethics approval

This study used *Caenorhabditis elegans*, an invertebrate model organism that is not subject to animal ethics committee oversight under current institutional and national guidelines.

## Conflict of interest

The authors declare no conflict of interest.

## Author contributions

Conceptualisation, BMcD, KW and QX; methodology, QX, JT, JC, PL, MB, LQ, KW and BMcD; investigation, QX, JT, JC, MB, PL and BMcD; resources, LQ, KW and BMcD; writing‐original draft, QX and BMcD; writing‐review and editing, QX, JT, PL, JC, LQ, KW and BMcD; supervision, LQ, KW and BMcD.

## Data Availability

Source data for the results presented in this manuscript are available at Mendeley data: doi: 10.17632/sj5bstcj4m.1.
